# A novel small molecule, AS1, reverses the negative hedonic valence of noxious stimuli

**DOI:** 10.1186/s12915-023-01573-7

**Published:** 2023-04-03

**Authors:** Kali Esancy, Lais L. Conceicao, Andrew Curtright, Thanh Tran, Logan Condon, Bryce Lecamp, Ajay Dhaka

**Affiliations:** 1 Department of Biological Structure, University of Washington, Seattle, USA; 2 Graduate Program in Neuroscience, University of Washington, Seattle, USA

**Keywords:** Pain, Aversion, Valence, Dopamine, Reward circuitry, Analgesic, Drug discovery, Zebrafish

## Abstract

**Background:**

Pain is the primary reason people seek medical care, with chronic pain affecting ~ 20% of people in the USA. However, many existing analgesics are ineffective in treating chronic pain, while others (e.g., opioids) have undesirable side effects. Here, we describe the screening of a small molecule library using a thermal place aversion assay in larval zebrafish to identify compounds that alter aversion to noxious thermal stimuli and could thus serve as potential analgesics.

**Results:**

From our behavioral screen, we discovered a small molecule, Analgesic Screen 1 (AS1), which surprisingly elicited attraction to noxious painful heat. When we further explored the effects of this compound using other behavioral place preference assays, we found that AS1 was similarly able to reverse the negative hedonic valence of other painful (chemical) and non-painful (dark) aversive stimuli without being inherently rewarding. Interestingly, targeting molecular pathways canonically associated with analgesia did not replicate the effects of AS1. A neuronal imaging assay revealed that clusters of dopaminergic neurons, as well as forebrain regions located in the teleost equivalent of the basal ganglia, were highly upregulated in the specific context of AS1 and aversive heat. Through a combination of behavioral assays and pharmacological manipulation of dopamine circuitry, we determined that AS1 acts via D1 dopamine receptor pathways to elicit this attraction to noxious stimuli.

**Conclusions:**

Together, our results suggest that AS1 relieves an aversion-imposed “brake” on dopamine release, and that this unique mechanism may provide valuable insight into the development of new valence-targeting analgesic drugs, as well as medications for other valence-related neurological conditions, such as anxiety and post-traumatic stress disorder (PTSD).

**Supplementary Information:**

The online version contains supplementary material available at 10.1186/s12915-023-01573-7.

## Background

Hedonic valence is a measurement of the intrinsic value of a stimulus and can be positive (attractive), negative (aversive), or neutral. Pain typically has a negative valence, which is normally advantageous, as it drives self-protective behavior. In chronic pain conditions, however, this ordinarily helpful sense becomes maladaptive, and the negative valence associated with these disordered affective states can fuel suffering. Conversely, humans can sometimes assign a positive valence to nociceptive stimuli, for example finding pleasure in spicy foods. This implies that the neural circuits that assign negative valence to nociceptive stimuli are malleable and that pain and aversion can be decoupled, providing a potential avenue for therapeutic intervention.

How motivational valence is assigned in the brain has been and continues to be the subject of much research and discussion [1–4]. In mammals the determination of aversive motivational valence has been attributed to a number of areas within the central nervous system (CNS) most notably the striatum and the amygdala [5–10]. With respect to painful stimuli, these areas as well as the anterior cingulate cortex (ACC), insula, the thalamus, habenula, hypothalamus, and brainstem nuclei including the parabrachial nuclei have also been implicated in assigning negative affect [11–17]. Dopaminergic signaling within the mesolimbic system has long been associated with reward or providing a positive valence for pleasurable stimuli. In the presence of noxious stimuli, the dopamine reward system is actively repressed, driving activation of circuits that promote aversion [18–20]. Intriguingly, activation of dopamine signaling has also been shown to have anti-nociceptive effects and to participate in endogenous analgesic pathways, for example stress-induced analgesia [21–24]. Despite great advances in understanding the neuronal circuits regulating pain sensation, however, there remain significant deficits in our understanding of how negative valence is attributed to noxious stimuli.

Zebrafish provide an attractive model system for inquiries into the biology of nociception. They can be generated in large numbers, have low maintenance costs, are easy to genetically manipulate, and their small size and optical clarity allow for large-scale behavioral analysis and whole nervous system activity profiling. The organization of peripheral and central nociceptive processing systems is remarkably similar between teleost fish such as zebrafish and other vertebrates such as rodents and humans [25–28]. Even at timepoints as early as 1–3 days post fertilization (dpf), we and others have shown that this nociceptive processing system is similarly organized and functional [26, 29–34]. While still developing, larval zebrafish are fully functioning animals, which must hunt for prey and assign the appropriate valence to salient stimuli in order to survive. Notably, anatomical and functional dopamine signaling pathways are conserved in larval zebrafish and subcortical structures of the zebrafish telencephalon and diencephalon analogous to the striatum, amygdala, hypothalamus, and habenula have been implicated in driving reward and aversion [35–44]. These findings suggest that the neural circuits underpinning the determination of appetitive or aversive valence are largely conserved between larval zebrafish and mammals.

To investigate how valence is assigned to nociceptive stimuli, we utilized an operant place aversion assay in larval zebrafish to screen a small molecule library to identify compounds that alter aversion to noxious thermal stimuli. Here we describe a small molecule, Analgesic Screen 1 (AS1), which remarkably reverses the valence of noxious stimuli, rendering them attractive. The effects of AS1 were dose-dependent such that an intermediate dose could erase the aversion to the noxious stimulus without evoking preference. These results suggest that the setting of valence (appetitive, neutral, or aversive) in larval zebrafish can be effectively tuned. Furthermore, AS1-induced attraction to noxious stimuli was directly proportional to the intensity of the noxious stimuli. We found that the effects of AS1 are dependent on dopaminergic signaling via D1 dopamine receptors, suggesting that AS1 elicits its effects in part by relieving an intensity-encoded pain-imposed “brake” on dopamine release. This is in contrast to addictive opioid analgesics such as morphine, which activate reward circuitry independent of context while simultaneously suppressing nociceptive circuitry.

## Results

### The novel analgesic AS1 reverses the valence of normally aversive stimuli

**Fig. 1:**
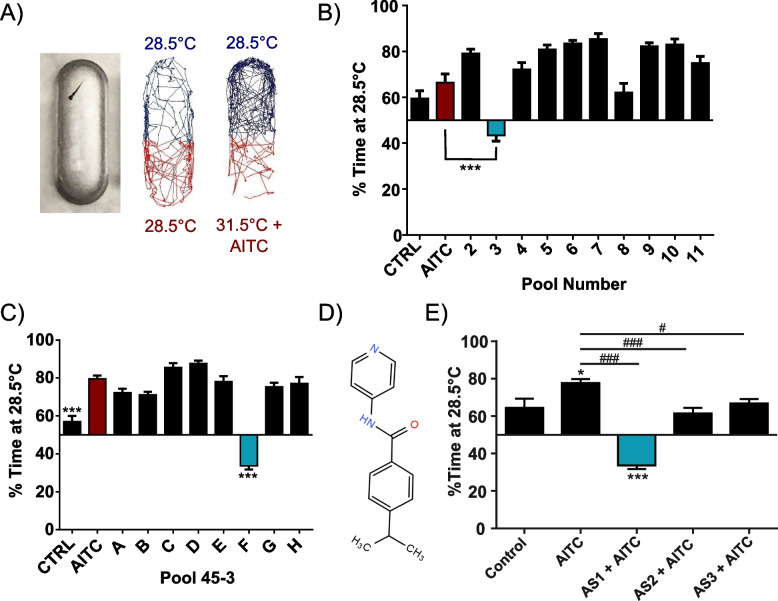
Identifying AS1, a novel compound that reverses sensitized thermal aversion, from a small molecule screen. **A** Left: photograph of a 5dpf zebrafish larva in a single arena of a 32-well temperature choice assay plate. Middle: representative traces/motion tracking of a zebrafish larva when both sides of the plate are set to 28.5 °C. No preference is demonstrated. Bottom: representative traces of a zebrafish larva given the choice between 28.5 and 31.5 °C after incubation in AITC. Clear preference for the 28.5 °C side is shown. **B** Results from screening Plate 43 in the sensitized temperature preference assay. Fish in all conditions except Pool 3 significantly favored the 28.5 °C side of the arena, whereas fish incubated in Pool 3 significantly chose the 31.5 °C side. *N* = 50 larvae for the control condition, *N* = 49 for the AITC only condition, *N* = 64, 52, 57, 63, 63, 61, 59, 63, 54, 58 larvae for Pools 2–11, respectively. **C** Demultiplexing Pool 45–3. Fish in all conditions except Pool F significantly preferred the 28.5 °C side, whereas fish in Pool F preferred the 37.5 °C side. *N* = 52, 63, 63, 63, 64, 58, 61, 64, 62, 57 larva for control, AITC, and groups A–H. *** denotes significant difference between the control and Pool F groups and the AITC only group. **D** The chemical structure of AS1 (4-propan-2-yl-N-pyridin-4-ylbenzamide). **E** Comparison of AS1 with two other potential novel analgesics, AS2 and AS3. *N* = 29, 58, 64, 49, 63 larvae for control, AITC, and AS1-3 conditions, respectively. * denotes significant difference from DMSO-treated control fish; # denotes significant difference from AITC-treated fish. *^/#^ *p* < 0.05, **^/##^ *p* < 0.01, ***^/###^ *p* < 0.001. One-way ANOVA with Dunnett’s multiple comparison tests was performed in **B** and **C**. One-way ANOVA with Tukey’s multiple comparisons test was performed in **E**. For all choice experiments (**B, C, E**), a one-sample *t* test was performed with a hypothetical mean of 50% to determine if fish were significantly choosing one side of the arena

In a previously published study, we developed a novel high-throughput temperature discrimination assay utilizing larval zebrafish that modeled acute and sensitized temperature aversion (Fig. 1A) [30]. Our assay revealed that larval zebrafish are exquisitely averse to temperatures that deviate from rearing temperature (28.5 °C) in a temperature-dependent manner. In the sensitized temperature aversion assay, larval zebrafish were pre-incubated in the inflammatory noxious chemical irritant and transient receptor potential ankyrin 1 (TRPA1) agonist allyl isothiocyanate (AITC) at a concentration that does not evoke locomotor escape behaviors (0.5 μM) [26, 45, 46]. When given a choice between their rearing temperature (28.5 °C) and mildly aversive heat (31.5 °C), larvae pre-incubated in this sub-threshold AITC concentration demonstrated greatly potentiated aversion to the 31.5 °C zone, mimicking conditions of thermal hyperalgesia. We went on to show that multiple small molecule analgesics could reverse acute and/or sensitized thermal aversion while non-analgesics could not. These studies established that thermal aversion in larval zebrafish is reflective of nociceptive behavior and that our assays could be an important tool in the identification of small molecules that disrupt or enhance nociceptive behavior and their cellular and molecular targets [30].

Using the sensitized thermal aversion assay, we screened a small molecule library of 3752 compounds in order to identify targets in pain transduction pathways and potential entry points for therapeutic intervention. The compounds were chosen from Chembridge’s CNS-Set, which consists of small molecules selected for blood brain barrier penetration and oral bioavailability. Compounds were picked from across the library to maximize the diversity of molecules screened. Small molecules were initially pooled (8 per pool) to maximize screening efficiency. Larvae (*n* ~ 64) in individual choice testing arenas were incubated in small molecule pools for 10 minutes min followed by the addition of AITC (0.5 μM), and then tested for thermal preference (28.5 vs 31.5 °C). We identified a pool from plate 45, column 3 that remarkably appeared to induce a slight preference to the noxious stimuli (Fig. 1B). Demultiplexing this pool identified one compound (4-propan-2-yl-*N*-pyridin-4-ylbenzamide, hereafter referred to as AS1), with no known prior biological activity, that strongly induced place preference for the noxious zone (Fig. 1C, D). In addition to AS1, we identified two other molecules (AS2 and AS3) that blocked the sensitized thermal aversion elicited by AITC (Fig. 1E). Notably, these two molecules, like previously tested analgesic compounds, only lessened aversion for the noxious zone, dramatically differing from the AS1-induced preference for noxious zone we observed [30]. This remarkably low hit rate (~ 0.08%) of compounds that had any effect upon thermal preference reflects the specificity of this assay and underscores the utility of high-throughput behavioral screens such as ours in the ongoing search for novel analgesics. Raw data from the screen is documented in Additional file 1: Database 1.

**Fig. 2:**
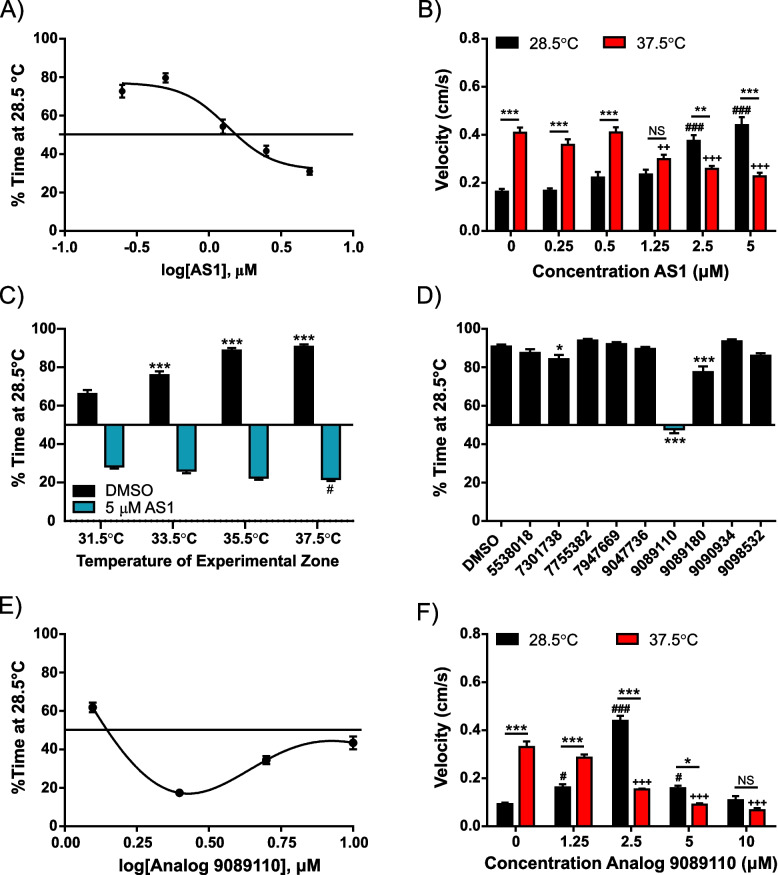
Probing the effects of AS1 using a non-sensitized thermal aversion assay. **A** AS1 dose–response curve in the non-sensitized thermal preference assay (28.5 vs 37.5 °C). At low concentrations, AS1 does not influence preference for 28.5 °C, whereas intermediate concentrations (1.25 μM) eliminate preference for either zone, and high concentrations (> 2.5 μM) induce preference for the 37.5 °C zone. *N* = 58, 58, 60, 63, 63, 62 for 0, 0.25, 0.5, 1.25, 2.5, and 5 μM AS1 conditions, respectively. **B** Velocity data for experiments shown in **A**. In control conditions (0 μM AS1), larval zebrafish locomote at much greater velocities in the 37.5 °C zone than in the 28.5 °C zone. Interestingly, as the concentration of AS1 increases, this velocity difference switches—velocity increases in the 28.5 °C portion but decreases in the 37.5 °C portion. At an intermediate concentration, there is no difference between velocities at 28.5 and 37.5 °C. * denotes significant differences in swimming velocities between the 28.5 and 37.5 °C zones for the same group of fish, + denotes significant difference from the 0 μM AS1 37.5 °C swimming velocity, and # denotes significant difference from the 0 μM AS1 28.5 °C swimming velocity. **C** Thermal preference assay assessing choice between rearing temperature (28.5 °C) and a range of aversive temperatures (31.5 °C, 33.5 °C, 35.5 °C, and 37.5 °C). As the intensity of the heat stimulus increases, vehicle-treated fish increasingly prefer the 28.5 °C zone, while the AS1-treated fish increasingly prefer the hot zone. All DMSO-treated fish significantly preferred the 28.5 °C, whereas all AS1-treated fish significantly preferred the 37.5 °C side, regardless of temperature comparison. *N* = 117 fish for DMSO and 127 for AS1 (31.5 °C), 111 for DMSO and 126 for AS1 (33.5 °C), 105 for DMSO and 125 for AS1 (35.5 °C), 73 for DMSO and 124 for AS1 (37.5 °C). * represents significant differences from 31.5 °C DMSO condition, whereas # represents significant differences from the 31.5 °C AS1 condition. **D** Thermal preference assay (28.5 °C vs 37.5 °C) utilizing nine different structural analogs of AS1. While two analogs slightly decreased the preference for 28.5 °C, only one of them (9089110) abolished preference for either side. *N* = 84, 59, 58, 46, 59, 55, 53, 32, 61, 59 fish for DMSO, 5538018, 7301738, 7755382, 7947669, 9047736, 9089110, 9089180, 9090934, and 9098532 conditions, respectively. **E** Analog 9089110 dose–response curve in the non-sensitized thermal preference assay (28.5 vs 37.5 °C). At low concentrations, Analog 9089110 does not influence preference for 28.5 °C, whereas higher concentrations (2.5 and 5 μM) induce preference for the 37.5 °C zone, and the highest concentration tested (10 μM) ablated choice. *N* = 103, 120, 119, 119, and 86 fish for 0, 1.25, 2.5, 5, and 10 μM Analog 9089110, respectively. **F** Velocity data for the experiments shown in **E**. As with AS1, in control (0 μM Analog 9089110) conditions, larval zebrafish locomote at significantly greater velocities in the 37.5 °C zone than the 28.5 °C zone. As the concentration of analog increases, zebrafish instead swim more quickly in the 28.5 °C zone and less quickly in the 37.5 °C zone, although at higher concentrations, zebrafish locomote slowly in both temperature zones, possibly due to motor impairment. */^#^/^+^ *p* < 0.05, **/^##^/^++^ *p* < 0.01, ***/^###^/^+++^ *p* < 0.001. A two-way ANOVA with Tukey’s multiple comparisons test was performed on the data in **B, C**. A one-way ANOVA with Tukey’s multiple comparisons test was performed on the data in **D**. A two-way ANOVA with Sidak’s multiple comparisons test was performed on the data in **F**. For thermal preference assays, a one-sample *t* test was performed with a hypothetical mean of 50% to determine if fish were significantly choosing one side of the arena over the other. Data shown in **A** was fit with the log(inhibitor) vs. response—Variable slope (four parameters) nonlinear fit option in GraphPad Prism, whereas the data in **E** was fit with the nonlinear bell-shaped dose–response curve function in GraphPad Prism

To determine if the effects of AS1 extended to acute noxious temperature sensation, we performed a dose–response analysis to determine the effects of AS1 on acute heat aversion (28.5 vs 37.5 °C). The highest dose of AS1 (5 μM) evoked strong preference for the nociceptive stimulus (Additional file 2: Movie 1). At lower doses, AS1 had intermediate effects, either reducing aversion or inducing a neutral response where the noxious stimulus was neither aversive nor attractive (Fig. 2A). Notably, AS1 also inverted zone-dependent locomotor activity when compared to vehicle-treated larvae in a dose-dependent manner, with an intermediate dose leading to an equalization of velocity in both zones. This implies that as the AS1 concentration increased, the 28.5 °C zone progressively became more aversive in addition to the 36.5 °C zone becoming more attractive (Fig. 2B). This dose dependency suggests that the setting of valence (appetitive, neutral or aversive) can be effectively tuned and that AS1 is acting upon a specific target. We additionally tested the effects of AS1 upon larval temperature preference between 28.5 °C and a range of aversive heat stimuli (31.5–37.5 °C). In vehicle-treated larvae, preference for the rearing temperature scaled with the intensity of the heat stimulus; i.e., larvae exhibited mild preference for 28.5 °C when given the choice between that and 31.5 °C, and stronger and stronger preference as the temperature of the experimental zone increased (Fig. 2C). Interestingly, the effects of AS1 also scaled with stimulus intensity—as the temperature of the experimental zone increased, AS1-treated fish demonstrated stronger preference for normally aversive noxious heat (Fig. 2C). This data suggest that valence is precisely encoded to relay the intensity of a heat stimulus and that AS1 can ablate aversion and induce preference in direct proportion to the strength of the heat stimulus.

To potentially identify the specific structural aspect responsible for AS1’s unique effects (which may yield some insight as to its molecular target), we performed our thermal aversion assay with structurally similar chemical analogs of AS1 (Fig. 2D, Additional file 3: Supplementary Table 1). Of the nine chemicals we tested (all at 5 μM, the maximally effective concentration of AS1), only one, Analog 9089110 (4-propyl-N-4-pyridinylbenzamide), completely ablated aversion to 37.5 °C, although at 5 μM it did not induce a strong preference for this lethal temperature. Intriguingly, when we later performed a dose–response analysis with this analog in our temperature choice assay, it was revealed that it may actually be slightly more potent than AS1, eliciting maximal attraction to 37.5 °C at 2.5 μM (Fig. 2E). Similarly to AS1, this compound also inverted locomotor activity, increasing swimming velocity at 28.5 °C and decreasing velocity at 37.5 °C (Fig. 2F). This inversion peaked at a dose of 2.5 μM, after which velocity in both zones notably diminished. This may be due to impairment of motor movements at high concentrations. The only structural difference between AS1 and Analog 9089110 is that the latter has a propyl group, as opposed to a propan-2-yl group, emerging from the benzene ring of the *N*-pyridin-4-ylbenzamide backbone that both of these molecules share. Further lengthening the alkyl group attached to the benzamide ring prevented an analog (Analog 9098532, or 4-butyl-N-4-pyridinylbenzamide) from replicating the effects of AS1 and Analog 9089110. Other minute changes in the structure of AS1 dramatically reduced its potency. For example, Analog 7301738 (4-isopropyl-N-3-pyridinylbenzamide) differs from AS1 only in the position of the nitrogen atom in the pyridine ring, yet it only had a small, if still significant, effect on temperature preference (Fig. 2D). The importance of this nitrogen atom is underscored by the fact that Analog 9090934 (4-propyl-N-3-pyridinylbenzamide), which is identical to Analog 9089110 except for the position of this atom, similarly has no effect upon temperature preference. Modifying the position and number of alkyl groups attached to the benzamide (i.e., in Analogs 7755382 and 9047736, 2,4-dimethyl-N-4-pyridinylbenzamide and 2,4,6-trimethyl-N-4-pyridinylbenzamide, respectively) likewise blocked any valence reversal effects. Intriguingly, the only other molecule that had a significant (if small) influence upon temperature choice behavior (Analog 9089180, or N-(4-isopropylphenyl)-3,5-dimethyl-4-isoxazolecarboxamide) exhibited the greatest level of structural difference from AS1 out of all of the molecules we tested. It is possible that this chemical is eliciting mild analgesia via an entirely different mechanism than that of AS1. Together, these results suggest that the overall chemical structure of AS1 is critical to elicit AS1’s molecular effects and may underlie its specificity.

**Fig. 3:**
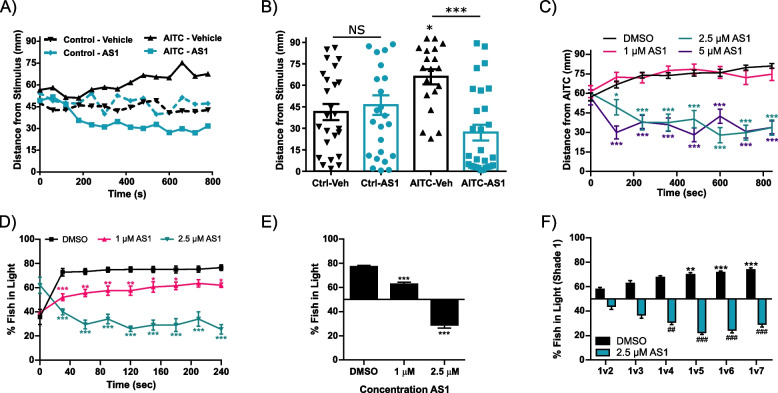
AS1 can reverse the valence of other sensory modalities. **A** Chemical attraction/aversion assay. When the experimental agarose is infused with 2% DMSO (dashed lines) both vehicle- and 5 μM AS1-treated fish demonstrate no preference for either end of the square chamber. When AITC (100 mM) is infused into the agarose (solid lines), the vehicle-treated fish are repelled by the agarose whereas the AS1-treated fish are attracted to the stimulus. *N* = 25 fish per condition. **B** The distance of each individual larva from the agarose at the final time point (*t* = 800 s) in **A**. In experiments with plain agarose, both AS1 and vehicle-treated fish are dispersed evenly throughout the arena. In experiments with AITC-infused agarose, vehicle-treated larval zebrafish are found significantly farther from the agarose while AS1-treated fish are clustered close to the AITC source. **C** AITC aversion assay assessing multiple concentrations of AS1. Fish exposed to low concentrations of AS1 (e.g., 1 μM) continue to avoid the AITC source, whereas fish exposed to a higher concentrations swim towards the AITC source. *N* = 83 larvae for DMSO, 42 for 1 μM, 33 for 2.5 μM, and 37 for 5 μM AS1. **D** Average percentage of fish on the light side of an arena in the phototaxis (light/dark preference) assay at 30-s intervals during a 4-minute trial period. Vehicle-treated fish strongly prefer (i.e., quickly swim towards) the light half of the arena, whereas fish treated with 2.5 μM AS1 quickly navigate towards the dark. *N* = 40 larvae per condition. **E** Same experiment as in **D**, but showing the percentage of fish found on the light side of the arena for the last 2 min of each 4-minute trial, averaged across the last four trials. While fish treated with vehicle and 1 μM AS1 significantly prefer the light side of the arena, fish treated with 2.5 μM significantly prefer the dark. **F** Gradient phototaxis assay in which larval zebrafish were given the choice between a bright white background (shade 1) and increasingly darker shades (shades 2–7). While all groups of DMSO-treated fish significantly prefer the light side and 2.5 μM AS1-treated fish significantly prefer the dark side, as the intensity of darkness increases, control zebrafish increasingly prefer shade 1, whereas 2.5 μM AS1-treated zebrafish increasingly prefer the contrasting dark shade. *N* = 72 for DMSO and 81 for AS1 (1v2), 73 for DMSO and 78 for AS1 (1v3), 80 for DMSO and 70 for AS1 (1v4), 79 for DMSO and 79 for AS1 (1v5), 79 for DMSO and 77 for AS1 (1v6), 76 for DMSO and 77 for AS1 (1v7). * represents significant difference from the DMSO 1v2 condition, whereas # represents significant difference from the AS1 1v2 condition. */^#^ *p* < 0.05, **/^##^ *p* < 0.01, ***/^###^ *p* < 0.001. One-way ANOVA with Tukey’s multiple comparison test was performed in **B, E**. Two-way ANOVA with Tukey’s multiple comparisons test was performed in **C**, **D**, **F**. For all temperature and light/dark choice experiments, a one-sample *t* test was performed with a hypothetical mean of 50% to determine if fish were significantly choosing one side of the arena

We next explored whether the effects of AS1 extended beyond noxious temperature sensation or could also impact other somatosensory modalities. To accomplish this, we adapted a chemical attraction/aversion assay where a thin layer of agarose was deposited along the edges of a square arena [47]. Along one wall, the agarose contained the chemical to be tested, while the other three walls were lined with control agarose. We anticipated that a chemical gradient would be established as chemical diffused from the agarose into the surrounding water. Larvae were pre-incubated in either vehicle or AS1 and then added to the arena and the distance of each larva from the chemical-infused agarose was measured. In control experiments where all four walls of the arena were lined with agarose only, AS1 and vehicle-treated larvae were dispersed evenly throughout the arena (Fig. 3A, B). This demonstrated that neither the arena nor AS1 itself induced preference/aversion for any side of the arena. When the nociceptive chemical AITC (100 mM, 10,000 × maximal effective dose) was tested, vehicle-treated larvae swam away from the AITC source. Remarkably, AS1-treated larvae strongly preferred to be near the AITC source, with the majority of larvae swimming directly to the AITC source, indicating attraction to this potently noxious nociceptive stimulus (Fig. 3A, B; Additional file 4: Movie 2; Additional file 5: Movie 3). Performing dose–response analysis revealed that 1 μM AS1 was unable to induce attraction to AITC whereas 2.5 μM AS1 largely replicated the effects of 5 μM AS1 (Fig. 3C).

Following this, we probed whether the effects of AS1 were restricted to somatosensation or were generalizable to other aversive stimuli. We thus tested the effects of AS1 on light/dark preference. Zebrafish larvae prefer white light environments to dark environments and this preference is inhibited by anxiolytics, analagous to studies in rodents [43, 48]. To measure light/dark preference, larval zebrafish were placed in a square arena and given the choice between a bright white light and total darkness, and the number of larvae on each side was quantified at 30-s intervals. Five 4-minute trials were performed, where the light and dark sides of the arena were reversed between trials. In alignment with our previous findings using heat and chemical stimuli, AS1 reversed light/dark preference in a dose-dependent manner, with AS1-treated larvae strongly preferring the dark environment while vehicle-treated fish preferred the light side of the chamber (Fig. 3D, E; Additional file 6: Movie 4). Given the precise control we had over stimulus luminosity in this assay, we next explored the effects of altering the intensity of the dark stimulus. In this gradient version of the phototaxis assay, larval zebrafish were given the choice between a bright white environment and one of six potential shades of darkness, ranging from light gray to solid black. Interestingly, the effects of AS1 scaled with the intensity of the dark stimulus inversely to our observation with control fish—while darker and darker shades of gray elicited greater and greater avoidance of the dark half of the arena in DMSO-treated fish, AS1-treated fish demonstrated greater and greater attraction to the dark (Fig. 3F). This implies that the valence of non-nociceptive aversive stimuli is also precisely encoded in an intensity-dependent manner, and that AS1 similarly exerts its effects in proportion to stimulus intensity. Collectively, these data suggest that AS1 can ablate aversion and instead induce preference for both nociceptive and other aversive stimuli.

### Canonical pain-relief circuitry is not involved in mediating the effects of AS1

**Fig. 4:**
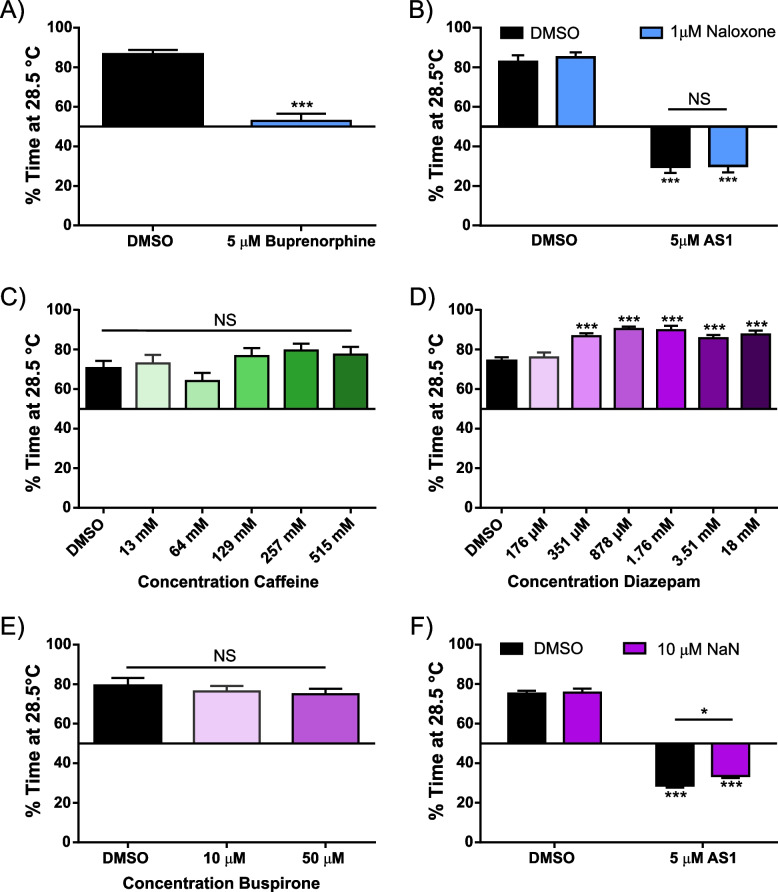
AS1 does not appear to act upon canonical valence or pain-relief circuitry. **A** Temperature choice assay (28.5 °C vs 37.5 °C) with buprenorphine, an opioid receptor agonist. While control fish significantly prefer the 28.5 °C zone, preference for either side is ablated in fish treated with 5 μM buprenorphine. *N* = 51 fish for both conditions. **B** Temperature choice assay (28.5 °C vs 37.5 °C) with naloxone, an opioid receptor antagonist. Naloxone does not appear to replicate or attenuate the effects of AS1; both control and naloxone only treated fish significantly choose the 28.5 °C side of the arena, whereas AS1 only and Naloxone + AS1-treated fish significantly choose the 37.5 °C side. *N* = 37, 56, 52, and 45 fish for DMSO, 1 μM Naloxone, 5 μM AS1, and 1 μM Naloxone + 5 μM AS1 conditions, respectively. **C** Temperature choice assay (28.5 °C vs 37.5 °C) with various concentrations of caffeine, an anxiogenic stimulant. At all concentrations tested, larval zebrafish significantly chose the 28.5 °C side of the arena. *N* = 52, 34, 51, 52, 34, and 38 fish for 0–515 mM caffeine, respectively. **D** Temperature choice assay (28.5 °C vs 37.5 °C) with diazepam, a GABA-modulating anxiolytic drug. At all concentrations tested, larval zebrafish significantly chose the 28.5 °C side of the arena. *N* = 115, 50, 53, 51, 49, 54, and 39 fish for 0–18 mM diazepam, respectively. **E** Temperature choice assay (28.5 °C vs 37.5 °C) with buspirone, a 5HT_1A_ receptor agonist and anxiolytic drug. At all concentrations tested, larval zebrafish significantly preferred the 28.5 °C side of the arena. *N* = 27, 43, 44, fish for 0, 10, and 50 μM buspirone conditions. **F** Temperature choice assay (28.5 °C vs 37.5 °C) with NaN-190, a 5HT_1A_ receptor antagonist. All AS1-treated fish significantly preferred the 37.5 °C side of the arena, while non-AS1 treated fish preferred the 28.5 °C side. *N* = 256 larvae for DMSO only, 132 for 10 μM NaN-190, 231 for 5 μM AS1, and 230 for 10 μM NaN-190 + 5 μM AS1. * *p* < 0.05, ** *p* < 0.01, *** *p* < 0.001. Two-tailed unpaired *t* test used in A. Two-way ANOVA with Tukey’s multiple comparisons test used in **B**, **F**. One-way ANOVA with Tukey’s multiple comparisons test used in C-E. For all temperature choice experiments, a one-sample *t* test was performed with a hypothetical mean of 50% to determine if fish were significantly choosing one side of the arena

**Fig. 5:**
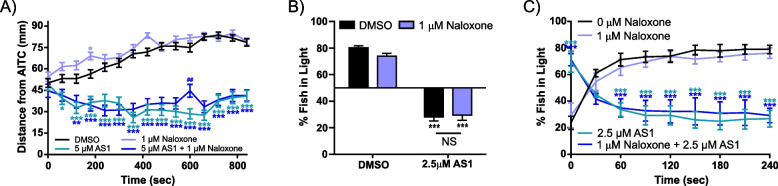
The opioid receptor antagonist naloxone does not replicate or reverse AS1-mediated attraction to noxious stimuli. **A** AITC aversion assay. Naloxone alone does not affect AITC avoidance, and when co-applied with AS1 this drug does not reverse AS1-induced attraction towards AITC. *N* = 80 fish for the DMSO condition, 71 for 5 μM AS1, 80 for 1 μM Naloxone, and 70 for 1 μM Naloxone + 5 μM AS1. * indicate significant difference from the DMSO control at each time point. **B** Phototaxis assay. All non-AS1-treated fish significantly chose the light side of the arena, whereas all AS1-treated fish significantly preferred the dark, regardless of whether 1 μM naloxone was applied. *N* = 39 fish for the DMSO condition, 41 fish for the 2.5 μM AS1 condition, 41 fish for 1 μM naloxone, and 39 fish for 1 μM naloxone + 2.5 μM AS1. **C** The percentage of fish in the light at 30-s intervals in the 4-min trials of the phototaxis assay shown in **B**, averaged across the five trials. * indicate significant difference from the DMSO (0 μM Naloxone) control at each time point. As shown, AS1-treated fish quickly migrate to the dark half of the arena at the onset of each trial, whereas fish that did not receive AS1 migrate to the light half of the arena, regardless of whether naloxone has been applied. * *p* < 0.05, ** *p* < 0.01, *** *p* < 0.001. Two-way ANOVA with Tukey’s multiple comparisons test used in **A–C**. To determine if fish were significantly choosing one side of the arena over the other a one-sample *t* test was performed with a hypothetical mean of 50%

Once we had established that AS1 could ablate aversion and induce preference for aversive stimuli across sensory modalities, we sought to discover the neural mechanisms underlying these effects upon hedonic valence. Given the wealth of literature upon opioid analgesics—which have anti-nociceptive properties and engage reward/valence circuitry—we explored the possibility of whether AS1 might be acting in a similar fashion. Both in vitro and in vivo studies suggest that zebrafish mu opioid receptor (MOR) has a pharmacological profile similar to that of mammalian MORs and that its activation elicits analogous physiological and behavioral effects [49, 50]. In line with our previous findings, where we demonstrated that buprenorphine acts as an analgesic in larval zebrafish in both sensitized and acute thermal preference assays, in our current study buprenorphine likewise reduced the amount of time larval zebrafish spent at 28.5 °C, but did not elicit an actual preference for noxious heat (Fig. 4A) [30]. Furthermore, naloxone, an OR antagonist, which we have previously shown reverses the analgesic effects of buprenorphine on thermal aversion, did not replicate or attenuate the effects of AS1 in the thermal preference, AITC aversion, or phototaxis assays (Fig. 4B, Fig. 5A–C) [30]. These data suggest that AS1 is acting upstream, or independently of, opioid signaling pathways to mediate its effects.

Curious, we then tested whether treatment with stimulants or anxiolytics could replicate the effects of AS1 in our thermal aversion assay. Some anxiolytics have been shown to have analgesic properties, and in zebrafish, can also attenuate light preference [43, 51]. Likewise, many stimulants have intrinsic analgesic properties, can potentiate the effects of opioid analgesia, and have been used by people as a self-medication strategy to treat chronic pain [52, 53]. Treatment with the stimulant/anxiogenic caffeine did not affect thermal preference for 28.5 °C at any of the concentrations we tested (Fig. 4C). Similarly, treatment with anxiolytic drugs (diazepam and buspirone) did not elicit attraction to lethal heat at any tested concentration. In fact, some concentrations of diazepam (a GABA receptor modulator) actually potentiated preference for 28.5 °C (Fig. 4D). Buspirone (a serotonin 5HT_1A_ receptor agonist) by contrast had no effect upon thermal preference (Fig. 4E). We also tested whether its antagonist NaN-190 could reverse the effects of AS1 (Fig. 4F). While we did observe a slight attenuation of AS1-induced preference for noxious heat at the highest concentration of NaN-190 we tested (10 μM), AS1-treated zebrafish still strongly preferred the hot zone over their rearing temperature, suggesting that AS1 does not act via this receptor to mediate valence reversal in the context of aversive stimuli (Fig. 4F).

**Fig. 6:**
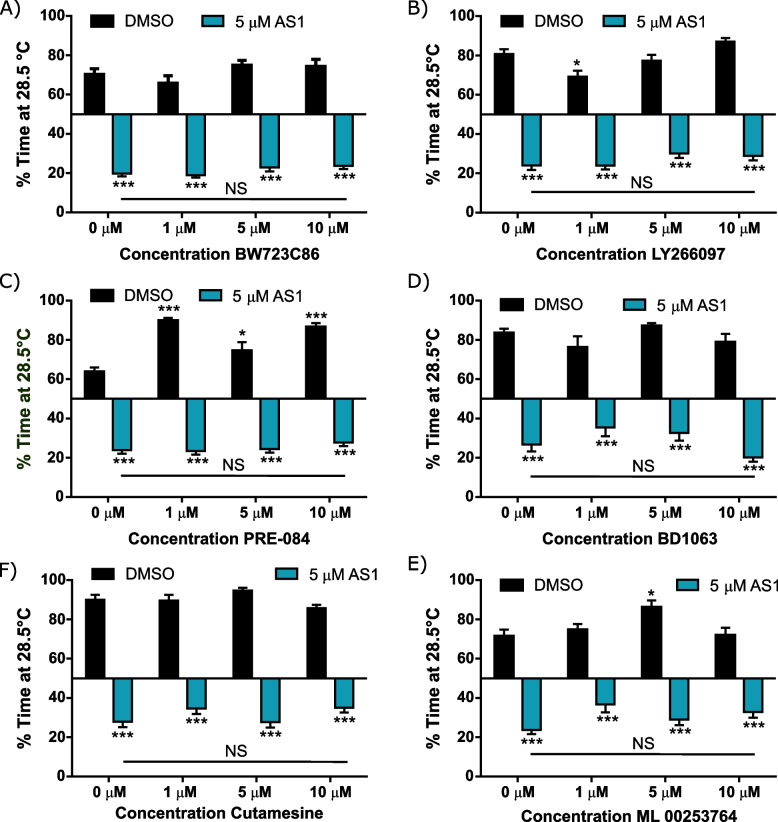
The 5HT_2B_, sigma-1, and melanocortin-4 receptors do not appear to mediate the effects of AS1. **A** Thermal preference assay (28.5 °C vs 37.5 °C) with the 5HT_2B_ agonist BW723C86. All AS1-treated fish significantly prefer the 37.5 °C half of the arena, regardless of whether BW723C86 was co-applied. All conditions that did not receive AS1 significantly chose the 28.5 °C side. At no concentration did BW723C86 influence AS1-induced attraction towards noxious heat. *N* = 46 fish for vehicle-treated controls, 43 for 1 μM BW723C86, 53 for 5 μM BW723C86, 53 for 10 μM BW723C86, 56 for 5 μM AS1, 50 for 1 μM BW723C86 + 5 μM AS1, 52 for 5 μM BW723C86 + 5 μM AS1, and 59 for 10 μM BW723C86 + 5 μM AS1. **B** Thermal preference assay (28.5 °C vs 37.5 °C) with the 5HT_2B_ antagonist LY266097. All AS1-treated fish significantly prefer the 37.5 °C half of the arena, regardless of whether LY266097 was co-applied. All conditions that did not receive AS1 significantly chose the 28.5 °C side. At no concentration did LY266097 influence AS1-induced attraction towards noxious heat. *N* = 58 larvae for the DMSO condition, 57 for 1 μM LY266097, 50 for 5 μM LY266097, 50 for 10 μM LY266097, 59 for 5 μM AS1, 61 for 1 μM LY266097 + 5 μM AS1, 59 for the 5 μM LY266097 + 5 μM AS1, and 56 for the 10 μM LY266097 + 5 μM AS1. **C** Thermal preference assay (28.5 °C vs 37.5 °C) with the Sigma-1 receptor agonist PRE-084. All AS1-treated fish significantly prefer the 37.5 °C half of the arena, regardless of whether PRE-084 was co-applied. All conditions that did not receive AS1 significantly chose the 28.5 °C side. At no concentration did PRE-084 influence AS1-induced attraction towards noxious heat, despite potentiating preference for the 28.5 °C zone. *N* = 90 fish for the DMSO condition, 27 for 1 μM PRE-084, 31 for 5 μM PRE-084, 25 for 10 μM PRE-084, 37 for 5 μM AS1, 40 for 1 μM PRE-084 + 5 μM AS1, 43 for 5 μM PRE-084 + 5 μM AS1, and 46 for 10 μM PRE-084 + 5 μM AS1. **D** Thermal preference assay (28.5 °C vs 37.5 °C) with the Sigma-1 receptor antagonist BD1063. All AS1-treated fish significantly prefer the 37.5 °C half of the arena, regardless of whether BD1063 was co-applied. All conditions that did not receive AS1 significantly chose the 28.5 °C side. At no concentration did BD1063 influence AS1-induced attraction towards noxious heat. *N* = 30 for the DMSO condition, 26 for 1 μM BD1063, 30 for 5 μM BD1063, 28 for 10 μM BD1063, 27 for 5 μM AS1, 30 for 1 μM BD1063 + 5 μM AS1, 29 for 5 μM BD1063 + 5 μM AS1, and 28 for 10 μM BD1063 + 5 μM AS1. **E** Thermal preference assay (28.5 °C vs 37.5 °C) with the sigma-1 receptor agonist cutamesine (SA 4503). All AS1-treated fish significantly preferred the 37.5 °C half of the arena, regardless of whether cutamesine was co-applied. All conditions that did not receive AS1 significantly chose the 28.5 °C side. At no concentration did cutamesine have any effect upon temperature preference when applied alone, nor did it significantly reduce AS1-induced attraction to noxious heat. *N* = 30 fish for the DMSO condition, 29 for 1 μM cutamesine, 25 for 5 μM cutamesine, 54 for 10 μM cutamesine, 52 for 5 μM AS1, 49 for 1 μM cutamesine + 5 μM AS1, 52 for 5 μM + 5 μM AS1, and 58 for 10 μM cutamesine + 5 μM AS1. **F** Thermal preference assay (28.5 °C vs 37.5 °C) with the melanocortin 4 receptor antagonist ML00253764. All AS1-treated fish significantly prefer the 37.5 °C half of the arena, regardless of whether ML00253764 was co-applied. All conditions that did not receive AS1 significantly chose the 28.5 °C side. At no concentration did ML00253764 reduce AS1-induced attraction towards noxious heat, even though one concentration (5 μM) did slightly potentiate preference towards the 28.5 °C zone. *N* = 30 fish for the DMSO condition, 31 for 1 μM ML00253764, 28 for 5 μM ML00253764, 31 for 10 μM ML00253764, 31 for 5 μM AS1, 32 for 1 μM ML00253764 + 5 μM AS1, 32 for 5 μM ML00253764 + 5 μM AS1, and 31 for 10 μM ML00253764 + 5 μM AS1. * *p* < 0.05, ** *p* < 0.01, *** *p* < 0.001. Two-way ANOVA with Sidak’s multiple comparisons test used for **A**; two-way ANOVA with Tukey’s multiple comparisons test used for **B–F**. To determine if fish were significantly choosing one side of the arena over the other, a one-sample *t* test was performed with a hypothetical mean of 50%

To further seek out the molecular target(s) of AS1, we utilized the resources of the Psychoactive Drug Screening Program (PDSP) [54]. Through this program, radioligand binding assays were used to determine whether AS1 interacted with any of a panel of 45 proteins found on neurons, including several serotonin, dopamine, opioid, adrenergic, and acetylcholine receptors, as well as neurotransmitter transporter proteins. Surprisingly, these assays identified no interaction between AS1 and the bulk of these receptors and proteins. Within this panel, only two had potential, if weak, interactions with AS1: the 5-HT_2B_ serotonin receptor and the sigma-1 receptor. While the Ki values of AS1 for each of these receptors were fairly high (1070.04 nM for 5HT_2B_ and 842.95 nM for sigma-1), indicating relatively low binding affinity, we still sought to verify whether these receptors were involved in mediating the effects of AS1. We once more employed our thermal preference assay, but following incubation in drugs that specifically acted upon these receptors. Treatment with the 5-HT_2B_ agonist BW723C86 did not replicate or reverse the effects of AS1 (Fig. 6A). Nor did treatment with the 5-HT_2B_ antagonist LY266097 replicate or reverse the effects of AS1 (Fig. 6B). Incubation in either the specific sigma-1 receptor agonist (PRE-084) or antagonist (BD1063) similarly yielded no effects (Fig. 6C, D). Intriguingly, activating the sigma-1 receptor has previously been shown to elicit a dramatic switch between passive (freezing) and active (escape) behavioral responses in zebrafish following exposure to an aversive strobe light stimulus [55]. To further confirm that the sigma-1 receptor was not involved in the AS1-induced behavioral switch in temperature preference, we repeated our experiment using the specific sigma-1 agonist cutamesine (also known as SA 4503) used in the freezing-to-escape behavior study (Fig. 6E) [55]. However, we found that treatment with cutamesine had no effect upon thermal preference either alone or when co-applied with AS1 (Fig. 6E). Together, these data suggest that AS1 is likely not acting on either of these PDSP-identified receptors to mediate its effects.

We additionally probed whether the melanocortin 4 receptor (MCR4), which has previously been implicated in valence reversal of nociceptive stimuli in rodents, could underlie the effects of AS1 [7, 56]. However, treatment with the MCR4 antagonist ML00253764 likewise did not replicate or attenuate AS1-induced attraction to noxious heat at any of the concentrations we tested (Fig. 6F). This suggests that unlike in rodents, MCR4 may not play a role in valence assignment in zebrafish, or at least that AS1 is not acting upon these receptors to elicit preference to aversive stimuli.

**Fig. 7:**
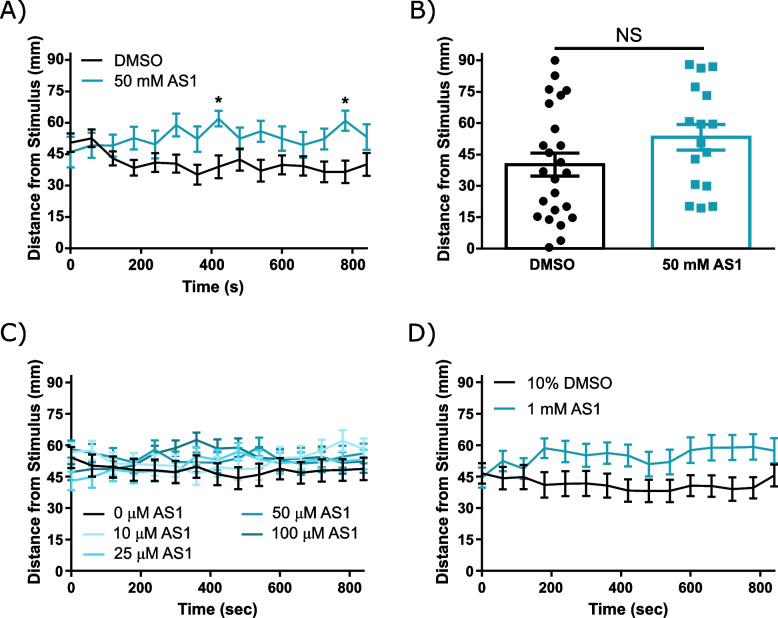
AS1 is not inherently attractive. **A** A chemical attraction/aversion assay in which either vehicle (2% DMSO) or 50 mM AS1 was infused into agarose lining one side of a square petri dish. As shown, larval zebrafish are not drawn towards the AS1 source. *N* = 27 larvae in the DMSO group, 16 in the AS1 group. **B** Distance of each individual larva from the AITC stimulus at the final time point (840 s) for the experiment shown in **A**.** C** Chemical attraction/aversion assay in which 2 mL of 1% DMSO or variable concentrations of AS1 (in 1% DMSO vehicle). Larval zebrafish do not appear to swim towards the AS1 source at any concentration tested for the duration of the assay. *N* = 36 fish for the DMSO condition, 36 for the 10 μM AS1 condition, 36 for the 25 μM AS1 condition, 40 for the 50 μM AS1 condition, and 40 for the 100 μM AS1 condition. **D** Same as **C**, but with 10% DMSO or 1 mM AS1 (in 10% DMSO). *N* = 36 larvae in both conditions. * *p* < 0.05, ** *p* < 0.01, *** *p* < 0.001. Two-tailed unpaired *t* test used in **B**. Two-way ANOVA with Sidak’s multiple comparisons test used in **A, D**. Two-way ANOVA with Tukey’s multiple comparisons test used in **C**

To confirm that AS1 itself was not attractive, we returned to our chemical attraction/aversion assay. When agarose containing AS1 at a concentration of 50 mM (10,000 × the effective dose) was deposited against one side of a square arena, larval zebrafish did not approach it in a way that was appreciably different from control agarose; if anything, AS1 appeared to be slightly aversive, although this was only significant at two time points (Fig. 7A, B). We also tested other concentrations of AS1 using an alternative attraction/aversion assay in which AS1 solutions were directly applied to the experiment chamber, rather than dissolved in agarose (see “Methods”). As expected based upon our initial results, AS1 continued to be neutral or mildly aversive for larval zebrafish even at higher concentrations (Fig. 7C, D).

Together, this data suggests that AS1 is acting to reverse valence via a unique molecular mechanism unlike those underlying traditional analgesics. Additionally, it does not possess any intrinsic attractiveness that have been observed in other drugs, such as opioid analgesics [57–59]. Instead, AS1 appears to elicit attraction only in the presence of a noxious stimulus, implying that activation of aversion-encoding neural circuitry is required for our observed hedonic shift.

### Brain regions associated with dopaminergic circuitry are specifically activated in the concurrent presence of AS1 and noxious stimuli

**Fig. 8:**
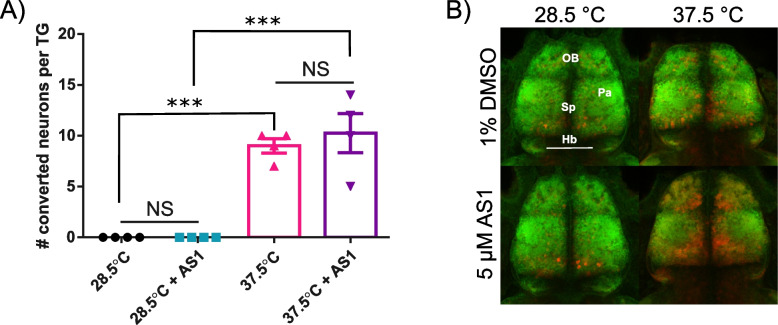
AS1 engages central nervous system circuitry in the presence of noxious stimuli. **A** Number of photoconverted trigeminal neurons in control or AS1-treated zebrafish exposed to either 28.5 or 37.5 °C. *N* = 4 larvae per condition. **B** Representative images of the telencephalon of 6dpf larval zebrafish exposed to 1% DMSO or 5 μM AS1, + / − noxious heat (37.5 °C) for 15 min and subsequently stained for pERK (red) and tERK (green). Images are cropped from maximum intensity projections (~ 35 μm) of confocal *z*-stacks taken of whole brains. *N* = 12 fish for DMSO + 28.5 °C, 10 fish for 5 μM AS1 + 28.5 °C, 14 fish for DMSO + 37.5 °C, and 19 fish for 5 μM AS1 + 37.5 °C. OB = olfactory bulb, Sp = subpallium, Pa = pallium, Hb = habenula. * *p* < 0.05, ** *p* < 0.01, *** *p* < 0.001. One-way ANOVA with Tukey’s multiple comparisons test used in **A**

We next sought to determine in an unbiased manner where in the zebrafish nervous system AS1 was exerting its effects by examining neuronal activity in the context of noxious stimuli. While the ability of AS1 to modify the valence of aversive stimuli across multiple sensory modalities implied that it likely acted via central nervous system mechanisms, our data did not rule out the possibility that peripheral nervous system mechanisms were also involved. Many analgesics can act upon multiple different levels of pain transduction circuitry. For example, MORs can be found upon peripheral somatosensory neurons, spinal cord neurons, and numerous neuronal populations in the brain, and both exogenous and endogenous opioids can modulate the activity of any of these neurons [60]. To investigate whether peripheral somatosensory neurons were also influenced by AS1, we performed a neuronal activity assay upon transgenic zebrafish expressing the genetically encoded calcium indicator CaMPARI in all neurons. This fluorescent protein permanently photoconverts from green to red in the presence of a 405-nm light and high calcium (a proxy for neuronal activity), allowing us to obtain “snapshots” of neuronal activity at a single time point [61]. We exposed fish to conditions of 28.5 or 37.5 °C with or without AS1 in the presence of a blue light, and then surveyed the trigeminal ganglia (TG) for photoconverted neurons. As expected, exposure to the rearing temperature of 28.5 °C did not elicit any conversion of trigeminal neurons in control zebrafish, whereas exposure to 37.5 °C led to the photoconversion of significantly more neurons (Fig. 8A). AS1-treated fish likewise exhibited an absence of TG neuronal activity under conditions of 28.5 °C and robust activity under exposure to noxious heat (Fig. 8A). No significant difference between AS1-treated and control fish were observed at either temperature. This data suggests that AS1 is not directly modulating the activity of peripheral heat-sensitive somatosensory neurons either by itself or in the presence of a nociceptive stimulus, and suggests that AS1 is likely acting downstream of these neurons.

To investigate how AS1 might alter central nervous system activity in the presence of nociceptive stimuli, we performed whole brain activity profiling with the neuronal activity marker phosphorylated ERK (pERK). Briefly, 6dpf larval zebrafish were exposed to noxious heat (37.5 °C) or rearing temperature (28.5 °C) in the presence of vehicle or AS1 (either 2.5 or 5 μM) for 15 min. We then performed immunolabeling to detect both total ERK (tERK) and pERK (Fig. 8B). Volumetric z-stacks of the entire brain of each fish were taken upon a confocal microscope, registered to a reference brain, and smoothed using an ImageJ script. We then used a previously established pipeline to quantify how neuronal activity was up- or downregulated in each annotated brain region in the zebrafish CNS between different groups of fish [62]. We initially compared brains from each experimental condition (AS1 + 28.5 °C, Vehicle + 37.5 °C, and AS1 + 37.5 °C) with Vehicle + 28.5 °C to determine how activity under these experimental conditions differed from the baseline state. We next subtracted the total change in signal in each brain region for AS1 + 28.5 °C and Vehicle + 37.5 °C treated fish from AS1 + 37.5 °C treated fish. This was done to look for brain regions specifically altered in the presence of the noxious stimulus and AS1, reasoning that these areas would drive attraction to noxious stimuli.

Strikingly, we found that in the presence of noxious heat and AS1 (5 μM), a large proportion of the most highly activated regions were located in the zebrafish subpallium, a broad telencephalic region that has been described as the equivalent of the mammalian basal ganglia, which includes the nucleus accumbens, striatum, and part of an extended amygdala (Table 1, Additional file 7: Supplementary Table 2) [63–66]. Dopaminergic regions, both within the subpallium and in the diencephalon (e.g., posterior tuberculum, hypothalamus), were also heavily represented (Table 1, Additional file 7: Supplementary Table 2). Additionally, diencephalic neuronal clusters classified by expression of genes required for dopaminergic development, such as Otpb and Isl1, were also active to a high degree (Table 1, Additional file 7: Supplementary Table 2). Intriguingly, AS1 does not appear to indiscriminately activate dopaminergic subpopulations—instead, only certain clusters appear to be recruited by the tandem application of AS1 and heat, and these clusters include those that project within and to the subpallium. Other highly active regions included the pallium, another broad telencephalic region that contains the teleost equivalent of the amygdala; neurons expressing Vmat2, a monoamine transporter; oxytocin (OXTL) neuronal clusters, which play roles in stress relief and nociception; telencephalic white matter tracts; other basal ganglia precursors such as the thalamic eminence; and Hcrt and Qrfp clusters, which are involved in arousal and motivation. Interestingly, in AS1-treated fish that were not exposed to noxious heat, most of these regions were not highly active—rather, AS1 alone primarily recruited neuron clusters within the mesencephalon and rhombencephalon (midbrain/hindbrain), although some diencephalic OXTL, Hcrt, and Qrfp clusters are still represented (Additional file 8: Supplementary Table 3). Larval zebrafish exposed only to noxious heat (without AS1) likewise failed to exhibit upregulation in forebrain regions associated with reward circuitry, arousal, and motivation; instead, heat exposure elicited activation primarily in hindbrain and spinal cord (Additional file 9: Supplementary Table 4). Findings were similar when larval zebrafish were treated with 2.5 μM AS1 (Additional file 10: Supplementary Table 5).

**Table 1:** Top 50 brain regions specifically upregulated in the context of 5 μM AS1 and noxious heat (37.5 °C)

ROI	Signal specific to AS1 + Heat
Telencephalon—Isl1 cluster 1	62,784.02
Telencephalon—Isl1 cluster 2	59,449.99
Telencephalon—Subpallial Otpb Cluster 2	57,862.31
Telencephalon—S1181t Cluster	55,333.89
Diencephalon—Eminentia Thalami	55,198.24
Telencephalon—Anterior Commisure	52,049.98
Telencephalon—Subpallial Gad1b cluster	51,995.37
Telencephalon—Olfactory bulb dopaminergic neuron areas	51,323.12
Telencephalon—Subpallium	50,437.3
Diencephalon—Otpb Cluster 2	48,466.37
Diencephalon—Retinal Arborization Field 4 (AF4)	47,600.85
Diencephalon—Oxtl Cluster 1 in Preoptic Area	47,413.8
Diencephalon—Retinal Arborization Field 2 (AF2- Approximate Location)	46,758.78
Diencephalon—Preoptic area Vglut2 cluster	46,521.07
Telencephalon—Vmat2 cluster	46,517.43
Telencephalon—Subpallial Otpb strip	45,224.41
Telencephalon—Subpallial dopaminergic cluster	44,557.21
Diencephalon—Retinal Arborization Field 3 (AF3)	43,716.94
Diencephalon—Dopaminergic Cluster 3—hypothalamus	40,019.12
Diencephalon—Isl1 cluster 1	39,266.09
Telencephalon -	38,670.72
Rhombencephalon—Glyt2 Cluster 8	38,553.58
Telencephalon—Subpallial Vglut2 Cluster	38,145.97
Diencephalon—Isl1 cluster 2	36,475.6
Telencephalon—Pallium	36,091.17
Diencephalon—Otpb Cluster 4	35,964.5
Diencephalon—Hypothalamus Qrfp neuron cluster	35,847.32
Rhombencephalon—Olig2 Cluster	35,771.76
Rhombencephalon—Gad1b Cluster 8	35,509.66
Diencephalon—Hypothalamus Hcrt Neurons	35,066.89
Telencephalon—Telencephalic Migrated Area 4 (M4)	35,038.42
Diencephalon—Oxtl Cluster 5	34,474.08
Diencephalon—Left Habenula Vglut2 Cluster	33,383.12
Rhombencephalon—Glyt2 Cluster 7	33,307.91
Diencephalon—Oxtl Cluster 2	32,549.34
Rhombencephalon—MiM1	32,356.03
Diencephalon—Dopaminergic Cluster 1—ventral thalamic and periventricular posterior tubercular DA neurons	31,653.23
Diencephalon—Hypothalamus s1181t Cluster	30,757.2
Telencephalon—Vglut2 rind	30,426.53
Mesencephalon—Vmat2 cluster2	28,483.57
Diencephalon—Olig2 Band 2	27,055.59
Rhombencephalon—Spiral Fiber Neuron Posterior cluster	26,252.82
Rhombencephalon—Mauthner	26,157.52
Diencephalon—Preoptic Area	26,037.78
Rhombencephalon—Gad1b Cluster 6	25,823.45
Telencephalon—Olig2 Cluster	25,415.34
Telencephalon—Olfactory Bulb	24,123.37
Diencephalon—Otpb Cluster 3	23,189.83
Rhombencephalon—Vglut2 cluster 1	23,112.53
Diencephalon—Ventral Thalamus	22,613.96

A list of the top 50 regions of interest (ROIs) identified from MAP-Mapping analysis of pERK/tERK-labeled brains of larval zebrafish that were incubated in 5 μM AS1 and exposed to noxious heat. The “Signal Specific to AS1 + Heat” was obtained by subtracting the net signals of the “AS1 Only” and “Heat Only” groups from the net signal of the “AS1 + Heat” group for each ROI. ROIs are ranked in order from highest to lowest signal

### AS1 specifically engages D1 receptor dopaminergic circuitry to mediate valence reversal

The enrichment of brain regions containing dopaminergic neurons or receiving dopaminergic innervation (i.e., clusters within the zebrafish basal ganglia equivalent) in the activity profiles of fish concurrently exposed to AS1 and noxious heat prompted us to further explore the hypothesis that AS1 engaged dopaminergic circuits. To accomplish this, we repeated our behavioral aversion assays following pharmacological manipulation of dopamine receptor signaling. Like mammals, zebrafish possess multiple dopamine receptors, and we targeted the analogs of mammalian receptors most associated with valence assignment, the D1 and D2 receptors [40, 67, 68]. In mammals, these dopamine receptor subtypes are largely expressed on non-overlapping populations of striatal medium spiny neurons (MSNs), which play opposing roles in valence assignment and reward processing—in brief, stimulation of D1R+ neurons has been shown to facilitate reward and positive valence assignment, whereas activation of D2R+ neurons elicits aversion and limits reward [69–71]. As a whole, dopamine release promotes reward, since it activates D1R+ neurons and inhibits D2R+ neurons via stimulation of their respective receptors.

**Fig. 9:**
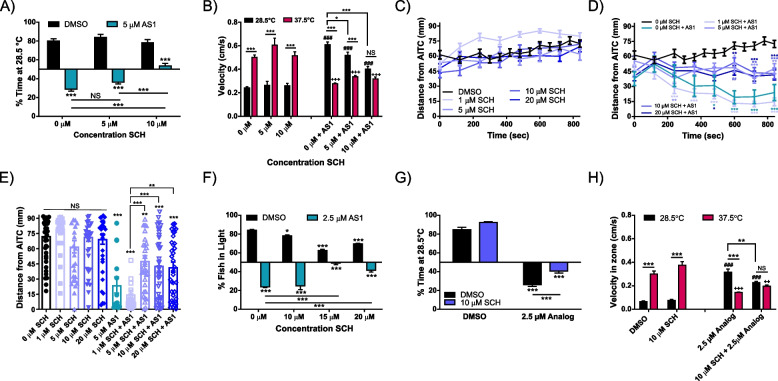
The D1 receptor antagonist SCH23390 partially reverses AS1-induced attraction to noxious stimuli. **A** Temperature choice assay (28.5 °C vs 37.5 °C) with SCH23390. Control fish and those treated with 5 or 10 μM SCH significantly preferred the 28.5 °C side of the arena, whereas fish treated with 5 μM AS1 alone or 5 μM SCH + 5 μM AS1 significantly preferred the 37.5 °C side. Interestingly, fish treated with 10 μM SCH + 5 μM AS1 exhibited no preference between the two sides of the arena, indicating an abolition of choice following D1 receptor blockade. *N* = 74 fish for the DMSO condition, 58 fish for 5 μM SCH, 43 fish for 10 μM SCH, 81 fish for 5 μM AS1, 52 fish for 5 μM SCH + 5 μM AS1, and 50 for 10 μM SCH + 5 μM AS1. **B** Velocity data for the experiment in **A**. While AS1 treatment still significantly reduces swimming velocity at 37.5 °C and increases velocity at 28.5 °C, treatment with progressively higher concentrations of SCH does significantly reduce this AS1-induced heightened velocity at 28.5 °C, and at 10 μM SCH (+ 5 μM AS1), there is no longer a significant difference in swimming velocity between the 28.5 and 37.5 °C zones. * denotes significant differences in swimming velocities between the 28.5 and 37.5 °C zones for the same group of fish, + denotes significant difference from the 0 μM AS1 37.5 °C swimming velocity, and # denotes significant difference from the 0 μM AS1 28.5 °C swimming velocity. **C, D** AITC aversion assay. SCH alone does not elicit changes in AITC avoidance, whereas concentrations of SCH 5 μM and above partially attenuate AS1-induced attraction to AITC. *N* = 44 fish for DMSO, 38 for 1 μM SCH, 34 for 5 μM SCH, 39 for 10 μM SCH, 37 for 20 μM SCH, 14 for 5 μM AS1, 43 for 1 μM SCH + 5 μM AS1, 33 for 5 μM SCH + 5 μM AS1, 41 for 10 μM SCH + 5 μM AS1, 41 for 20 μM SCH + 5 μM AS1. * represent the significant difference of experimental traces from the DMSO-treated control fish at each indicated time point. **E** Distance of each individual larva from the AITC stimulus at the final time point (840 s) for the experiment shown in **C, D**. * presented directly over columns represent the significant difference between the DMSO-only control condition, and between other columns when indicated by lines. **F** Phototaxis assay. While SCH alone does cause a significant decrease in the percentage of fish found in the light half of the arena when applied at higher concentrations, fish in these conditions still significantly prefer the light half of the arena. When co-applied with AS1, 15 μM and 20 μM SCH are able to significantly reduce AS1-induced preference for the dark, with 15 μM SCH abolishing preference entirely. *N* = 80 fish for DMSO, 80 for 10 μM SCH, 40 for 15 μM SCH, 80 for 20 μM SCH, 80 for 2.5 μM AS1, 40 for 10 μM SCH + 2.5 μM AS1, 40 for 15 μM SCH + 2.5 μM AS1, and 40 for 20 μM SCH + 2.5 μM AS1. **G** Temperature choice assay (28.5 °C vs 37.5 °C) with fish treated with 2.5 μM Analog 9089110 and/or 10 μM SCH23390. Control fish and those treated with 10 μM SCH significantly preferred the 28.5 °C side of the arena, whereas analog-treated fish significantly preferred the 37.5 °C side. As with AS1, treatment with 10 μM SCH23390 significantly attenuated analog-induced attraction to noxious heat, although these fish still preferred noxious heat over rearing temperature. *N* = 48 fish for the DMSO condition, 42 fish for 10 μM SCH, 55 fish for 2.5 μM Analog 9089110, and 60 fish for 2.5 μM Analog 9089110 + 10 μM SCH. **H** Velocity data for the experiment shown in **G**. * denotes significant differences in swimming velocities between the 28.5 and 37.5 °C zones for the same group of fish, + denotes significant difference from the 0 μM Analog 37.5 °C swimming velocity, and # denotes significant difference from the 0 μM Analog 28.5 °C swimming velocity. Similar to our AS1 experiments, treatment with 10 μM SCH was able to partially reverse the Analog-induced inversion of swimming velocity, at least in the 28.5 °C zone. */^#^/^+^ *p* < 0.05, **/^##^/^++^ *p* < 0.01, ***/^###^/^+++^ *p* < 0.001. Two-way ANOVA with Tukey’s multiple comparisons test used in **A–D, F–H**. One-way ANOVA with Tukey’s multiple comparisons test used in **E**. For all temperature and light/dark choice experiments, a one-sample *t* test was performed with a hypothetical mean of 50% to determine if fish were significantly choosing one side of the arena over the other

Remarkably, inhibition of D1 activity with the selective D1 antagonist SCH23390 (10 μM) ablated aversion to noxious heat in the presence of AS1 without eliciting effects at baseline (Fig. 9A). Additionally, SCH23390 was able to decrease the heightened velocity at 28.5 °C induced by AS1 in a dose-dependent manner. However, SCH23390 did not affect the AS1-evoked decrease in velocity at 37.5 °C (Fig. 9B). While SCH23390 by itself had no effect upon aversive behavior in response to the chemical irritant AITC at multiple concentrations, it ablated AS1-mediated attraction to AITC in a dose-dependent manner (Fig. 9C–E). These effects extended to the light–dark preference assay, with intermediate-high doses blocking AS1-induced preference for the dark (Fig. 9F). While high concentrations of SCH23390 do appear to significantly decrease light preference in the absence of AS1, it seems unlikely that this would explain the attenuation of the AS1-induced dark preference we observed following treatment with SCH23390, because such a shift is not opposing the effects of AS1. However, we cannot exclude the possibility that SCH23390-mediated D1 inhibition may be blunting phototaxic choice in general.

Application of SCH23390 similarly attenuated the effects of the AS1 Analog 9089110 in our thermal preference assay (Fig. 9G, H). While 10 μM SCH23390 had no effect on thermal preference in isolation, it was able to significantly reduce preference for 37.5 °C in fish that were also incubated in 2.5 μM Analog 9089110 (Fig. 9G). Likewise, SCH23390 was able to significantly reduce the analog-mediated heightened velocity at 28.5 °C, but unable to restore velocity in the 37.5 °C zone back to control levels (Fig. 9H). This implies that these structurally similar compounds likely act via similar mechanisms, both of which involve D1R circuitry.

**Fig. 10:**
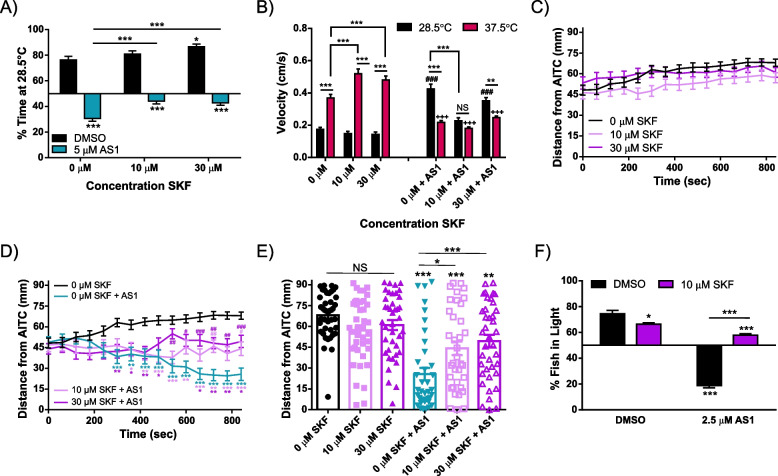
The D1 receptor agonist SKF82958 partially reverses AS1-induced attraction to noxious stimuli. **A** Temperature choice assay (28.5 °C vs 37.5 °C) with various concentrations of SKF82958. DMSO or SKF alone treated fish demonstrated significant preference for the 28.5 °C side of the arena, with the highest concentration of SKF tested eliciting a slight potentiation of preference for the 28.5 °C side. While all AS1-treated fish still preferred the 37.5 °C side of the arena regardless of whether SKF was also present, application of both 10 and 30 μM SKF did induce a significant decrease in the proportion of time fish spent in the 37.5 °C half of the arena. *N* = 53 fish for the DMSO condition, 53 for 10 μM SKF, 54 for 30 μM SKF, 61 for 5 μM AS1, 60 for 10 μM SKF + 5 μM AS1, and 60 for 30 μM SKF + 5 μM AS1. **B** Swimming velocity from the experiment shown in **A**. While AS1 treatment still reverses velocity patterns observed in the 37.5 and 28.5 °C zones, co-incubation with SKF partially attenuates this effect. At 10 μM SKF, the difference between swimming velocity in both zones is insignificant, and the swimming velocity at 28.5 °C is no different than that of vehicle-treated control fish. * denotes significant differences in swimming velocities between the 28.5 and 37.5 °C zones for the same group of fish, + denotes significant difference from the 0 μM AS1 37.5 °C swimming velocity, and # denotes significant difference from the 0 μM AS1 28.5 °C swimming velocity. **C, D** AITC aversion assay. SKF alone does not elicit any large changes in avoidance of the noxious AITC stimulus, but does partially reverse AS1-induced attraction towards AITC. *N* = 40 fish for the DMSO condition, 40 for the 10 μM SKF, 40 for the 30 μM SKF, 38 for 2.5 μM AS1, 36 for 10 μM SKF + 2.5 μM AS1, and 39 for 30 μM SKF + 2.5 μM AS1. * represent the significant difference of experimental traces from the DMSO-treated control fish at each indicated time point. # represent the significant difference of experimental traces compared to AS1-only control fish at the indicated time points. **E** Distance of each individual larva from the AITC source at the final time point (840 s) for the experiment shown in **C, D**. While AS1-treated fish cluster near the AITC source, fish co-incubated in SKF exhibit greater dispersal across the arena by the end of the experiment. * presented directly over columns represent the significant difference between the DMSO-only control condition, and between other columns when indicated by lines. **F** Phototaxis assay. While 10 μM SKF did elicit a slight decrease in the proportion of fish found in the light by itself, these fish still significantly preferred the light side, as did the vehicle-treated fish. AS1-only treated fish significantly preferred the dark half of the arena, but concurrent treatment with 10 μM SKF restored preference for the light side. *N* = 40 for all conditions. */^#^/^+^ *p* < 0.05, **/^##^/^++^ *p* < 0.01, ***/^###^/^+++^ *p* < 0.001. Two-way ANOVA with Tukey’s multiple comparisons test used in **A–D, F**. One-way ANOVA with Tukey’s multiple comparisons test used in **E**. For all temperature and light/dark choice experiments, a one-sample *t* test was performed with a hypothetical mean of 50% to determine if fish were significantly choosing one side of the arena over the other

We then reasoned that activating D1 receptors should also dampen but not reverse the effects of AS1 in these place preference assays, as D1 receptors would no longer be selectively activated in the presence of noxious stimuli. Indeed, application of the selective D1 receptor agonist SKF82958 blunted AS1-evoked preference for aversive stimuli in all assays. Both 10 μM and 30 μM SKF significantly attenuated the amount of time AS1-treated fish spent at 37.5 °C in the temperature preference assay (Fig. 10A). Much like SCH23390, SKF82958 also reduced the swimming velocity of AS1-treated zebrafish in the 28.5 °C zone, although swimming velocity in the 37.5 °C zone remained unaffected (Fig. 10B). SKF82958 by itself had little effect upon aversion to AITC (Fig. 10C, E), but did block AS1-induced preference to this chemical irritant (Fig. 10D, E). Similarly, exposure to the D1 receptor agonist modestly restored light preference in fish that were treated with AS1, although not to the level of vehicle-treated fish (Fig. 10F).

Interestingly, treatment with the D1R agonist was not able to completely ablate the effects of AS1 and restore thermal, chemical, and dark aversion back to baseline levels. At most, only weak aversion or neutral preference was observed, even at the highest concentrations of SCH23390 tested. Additionally, treatment with SCH was unable to reverse the AS1-induced reduction in swimming velocity at 37.5 °C. It is possible that AS1 may elicit analgesia separately from its effects upon valence assignment, and via a dopamine-independent mechanism.

**Fig. 11:**
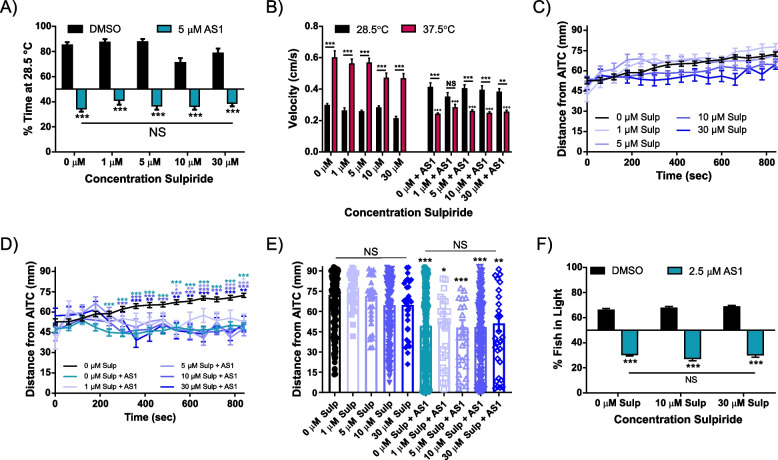
The D2 receptor antagonist sulpiride does not replicate or reverse the effects of AS1 in multiple choice assays. **A** Temperature choice assay (28.5 °C vs 37.5 °C) with various concentrations of sulpiride. All AS1-treated fish significantly preferred the 37.5 °C side, whereas fish treated only with sulpiride or vehicle solution significantly chose the 28.5 °C side. *N* = 28 larvae for 1% DMSO, 37 for 1 μM sulpiride, 41 for 5 μM sulpiride, 44 for 10 μM sulpiride, 30 for 30 μM sulpiride, 58 for 5 μM AS1, 49 for 1 μM sulpiride + 5 μM AS1, 52 for 5 μM sulpiride + 5 μM AS1, 54 for 10 μM sulpiride + 5 μM AS1, and 62 for 30 μM sulpiride + 5 μM AS1. **B** Swimming velocity of the fish in the experiment shown in **A**. AS1-treated fish have significantly lower velocities at 37.5 °C than at 28.5 °C at all concentrations of sulpiride tested apart from 1 μM. Additionally, AS1-treated fish have significantly lower swimming velocities at 37.5 °C than non-AS1 treated fish at all concentrations of sulpiride tested. * denotes significant differences in swimming velocities between the 28.5 and 37.5 °C zones for the same group of fish, + denotes significant difference from the 0 μM AS1 37.5 °C swimming velocity, and # denotes significant difference from the 0 μM AS1 28.5 °C swimming velocity. **C** AITC aversion assay with various concentrations of sulpiride. None of the sulpiride concentrations tested elicited a change in AITC aversion. *N* = 174 fish for DMSO, 36 fish for 1 μM sulpiride, 34 fish for 5 μM sulpiride, 177 fish for 10 μM sulpiride, and 33 fish for 30 μM sulpiride. **D** AITC aversion assay for various concentrations of sulpiride + 5 μM AS1, with DMSO control fish shown in **C**. At no concentration tested does sulpiride attenuate AS1-induced attraction to AITC. *N* = 169 fish for 5 μM AS1, 32 fish for 1 μM sulpiride + 5 μM AS1, 32 fish for 5 μM sulpiride + 5 μM AS1, 172 fish for 10 μM sulpiride + 5 μM AS1, and 33 fish for 30 μM sulpiride + 5 μM AS1. * represent the significant difference of experimental traces from the DMSO-treated control fish at each indicated time point. **E** The distance of each individual larva from the AITC-infused agarose at the final time point (*t* = 840 s) of the experiment shown in **C** and **D**. Whereas control and all sulpiride-only condition fish localize farther from the AITC source, all AS1-treated fish congregate closer to the AITC source. * presented directly over columns represent the significant difference between the DMSO-only control condition, and between other columns when indicated by lines. **F** Light/dark preference assay. No tested concentration of sulpiride was capable of reversing AS1-induced preference for the dark. *N* = 39 fish for 0 μM sulpiride, 40 for 10 μM sulpiride, 40 for 30 μM sulpiride, 77 for 0 μM sulpiride + 5 μM AS1, 79 for 10 μM sulpiride + 5 μM AS1, and 83 for 30 μM sulpiride + 5 μM AS1. */^#^/^+^ *p* < 0.05, **/^##^/^++^ *p* < 0.01, ***/^###^/^+++^ *p* < 0.001. Two-way ANOVA with Tukey’s multiple comparisons test used in **A–D, F**. One-way ANOVA with Tukey’s multiple comparisons test used in **E**. For all temperature and light/dark choice experiments, a one-sample *t* test was performed with a hypothetical mean of 50% to determine if fish were significantly choosing one side of the arena

**Fig. 12:**
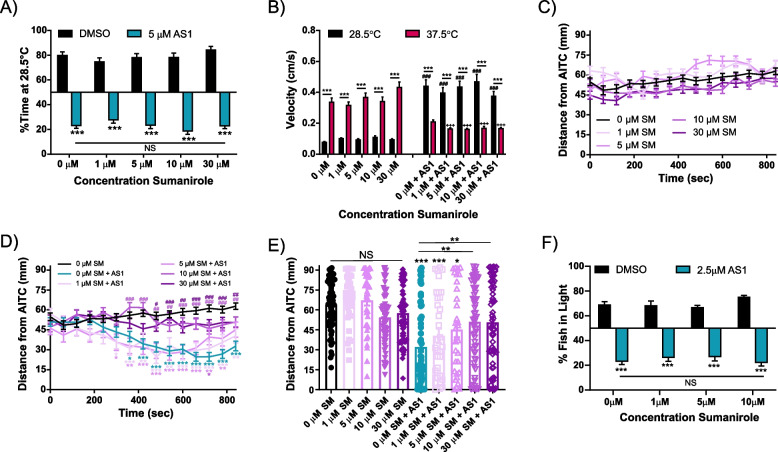
The D2 receptor agonist sumanirole maleate does not replicate or reverse the effects of AS1 in multiple assays. **A** Temperature choice assay (28.5 °C vs 37.5 °C) with various concentrations of sumanirole. All AS1-treated fish significantly preferred the 37.5 °C side regardless of sumanirole concentration, whereas fish treated only with sumanirole or vehicle solution significantly chose the 28.5 °C side. *N* = 44 fish for DMSO, 41 for 1 μM sumanirole, 59 for 5 μM sumanirole, 44 for 10 μM sumanirole, 48 for 30 μM sumanirole, 28 for 5 μM AS1, 46 for 1 μM sumanirole + 5 μM AS1, 50 for 5 μM sumanirole + 5 μM AS1, 37 for 10 μM sumanirole + 5 μM AS1, and 53 for 30 μM sumanirole + 5 μM AS1. **B** Velocity data for fish in the experiment shown in **A**. AS1 treatment significantly increases swimming velocity in the 28.5 °C zone and decreases swimming velocity in the 37.5 °C zone, regardless of the concentration of sumanirole co-applied to the zebrafish. Sumanirole alone has no effect upon swimming velocity in either zone. * denotes significant differences in swimming velocities between the 28.5 and 37.5 °C zones for the same group of fish, + denotes significant difference from the 0 μM AS1 37.5 °C swimming velocity, and # denotes significant difference from the 0 μM AS1 28.5 °C swimming velocity. **C, D** AITC aversion assay. Sumanirole alone does not affect aversion to this noxious chemical stimulus, but at higher concentrations partially attenuates AS1-induced attraction to AITC. *N* = 68 for 0 μM sumanirole, 36 for 1 μM sumanirole, 36 for 5 μM sumanirole, 61 for 10 μM sumanirole, 63 for 30 μM sumanirole, 64 for 0 μM sumanirole + 2.5 μM AS1, 34 for 1 μM sumanirole + 2.5 μM AS1, 34 for 5 μM sumanirole + 2.5 μM AS1, 61 for 10 μM sumanirole + 2.5 μM AS1, and 63 for 30 μM sumanirole + 2.5 μM AS1. * represent the significant difference of experimental traces from the DMSO-treated control fish at each indicated time point. # represent the significant difference of experimental traces compared to AS1-only control fish at the indicated time points. **E** Distance of each individual larva from the AITC stimulus at the final time point (840 s) from the experiment shown in **C**, **D**. * presented directly over columns represent the significant difference between the DMSO-only control condition, and between other columns when indicated by lines. **F** Phototaxis assay. AS1-treated fish significantly chose the dark half of the arena regardless of what concentration of sumanirole was co-applied, whereas vehicle and sumanirole-only treated fish significantly chose the light half of the arena. *N* = 23 fish for 0 μM sumanirole, 37 for 1 μM sumanirole, 41 for 5 μM sumanirole, 40 for 10 μM sumanirole, 40 for 0 μM sumanirole + 2.5 μM AS1, 46 for 1 μM sumanirole + 2.5 μM AS1, 43 for 5 μM sumanirole + 2.5 μM AS1, and 40 for 10 μM sumanirole + 2.5 μM AS1. */^#^/^+^ *p* < 0.05, **/^##^/^++^ *p* < 0.01, ***/^###^/^+++^ *p* < 0.001. Two-way ANOVA with Sidak’s multiple comparisons test used in **A, B**. Two-way ANOVA with Tukey’s multiple comparisons test used in **C, D, F**. One-way ANOVA with Tukey’s multiple comparisons test used in **E**. For all temperature and light/dark choice experiments, a one-sample *t* test was performed with a hypothetical mean of 50% to determine if fish were significantly choosing one side of the arena over the other

Treatment with the selective D2 receptor antagonist sulpiride alone had no effect upon the behavior of 6dpf larval zebrafish in the temperature choice assay, AITC aversion assay, or light/dark preference assay (Fig. 11A–F). Unlike the D1 receptor antagonist, application of sulpiride to AS1-treated fish had no effect upon AS1-induced reversal of temperature choice, swimming velocity in the 37.5 °C zone, AITC aversion, or light preference (Fig. 11A–F). Similarly, the selective D2 receptor agonist sumanirole maleate had no effect either alone or upon AS1-mediated attraction to noxious heat or dark stimuli (Fig. 12A, B, F). Intriguingly, while this drug had little effect alone in the chemical aversion assay, it did seem to attenuate AS1-mediated attraction to AITC, but only at higher concentrations (Fig. 12C–E). It is possible that in this particular scenario, activating the D2 receptor is mimicking the effects of SKF—by activating the D2 receptor, we are likely inhibiting neurons in the “off” pathway, perhaps creating the sensation of reward regardless of environmental context.

## Discussion

This work validates the use of larval zebrafish thermal aversion to identify compounds with analgesic potential. Here, we describe the identification of a small molecule, AS1, that reverses the hedonic valence of noxious stimuli. Further investigation revealed that this valence reversal was not limited solely to nociception, but was instead generalizable to other aversive conditions, such as dark environments. That we observed a clear dose–response relationship in our thermal and light/dark preference assays (i.e., as the concentration of AS1 was increased, the normally aversive 37.5 °C zone became neutral and then attractive) implies that the setting of valence is tunable. This in itself could inform future pain management strategies—if the negative valence responsible for the much of the suffering associated with pain can be pharmacologically tuned, then an analgesic could “dial down” this distress to neutral levels. Interestingly, the effects of AS1 scaled with stimulus intensity in both the thermal preference and light/dark preference assays. While increases in temperature or darkness elicited increased avoidance behaviors in vehicle-treated fish, AS1-treated fish demonstrated greater and greater preference in direct proportion to changes in noxiousness. This suggests that the larval zebrafish nervous system can precisely encode valence in order to relay the strength of negative stimuli and that AS1 acts upon such circuitry in an intensity-dependent manner.

Intriguingly, AS1 does not appear to modulate opioid receptor signaling. While buprenorphine, an opioid receptor agonist, was able to significantly reduce preference for 28.5 °C in our thermal preference assay, it did not induce attraction towards the 37.5 °C zone. Treatment with naloxone, an opioid receptor antagonist, did not attenuate the effects of AS1 in any of our assays, supporting the conclusion that AS1 does not directly influence opioid pathways. This is comparable to our previous investigations exploring the effects of analgesic compounds (e.g., clonidine, amitryptiline, TC-N 1752, HC-030031) upon zebrafish behavior in thermal preference assays—while many were capable of reducing thermal place preference, none elicited attraction to noxious heat [30]. This suggests that the various molecular targets of these drugs (the α2-adrenergic receptor, a serotonin and norepinephrine transport inhibitor, the nociceptor-specific sodium ion channel NaV_1.7_, and the receptor TRPA1) are unlikely to underlie AS1’s mechanistic action [30].

Furthermore, treatment with various anxiolytics (the GABA receptor modulator diazepam and the 5-HT_1A_ partial agonist buspirone) and caffeine (a stimulant) failed to replicate AS1-induced valence reversal in our thermal preference assays, suggesting that AS1 does not act upon these molecular targets. It is intriguing that these anxiolytics and stimulants did not affect temperature preference in our assays, despite previous reports in the literature demonstrating their clear impact upon light/dark preference, especially given that AS1 is capable of reversing valence across sensory modalities [43, 72]. There may be multiple potential mechanisms underlying this observation. A simple explanation is that aversion towards the dark and aversion towards painful somatosensory stimuli employ different neural circuits and molecular receptors. Dark avoidance in larval zebrafish is interpreted as an anxiety or fear-like behavioral response, perhaps as a strategy to avoid predators, whereas somatosensory stimuli such as extreme temperatures are avoided because they are nociceptive [72]. If only the dark avoidant circuits require receptors canonically involved relieving fear/anxiety (such GABA_A_ and 5-HT_1A_ receptors), then it is plausible that treatment with the anxiolytics that target these receptors could attenuate light preference but not heat preference. AS1 may then act independently upon each of these parallel circuits to attenuate choice behaviors across modalities. Alternatively, one could imagine a more complicated scenario in which information about dark and painful environmental stimuli could feed into the same neural circuits that establish general aversion. Even if this were the case, if the target receptors of diazepam and buspirone are only necessary at the nodes at which the pathways containing information about dark anxiety enter the general aversion circuits, one could logically expect that these drugs could attenuate light preference without affecting temperature preference. While we currently do not have concrete experimental evidence about AS1’s specific site of action, one might speculate that AS1 could attenuate aversion to both modalities by acting upon a different molecular target(s) at the point in aversion circuitry where these two information streams converge. Further studies are required to determine whether either of these scenarios, or an alternate mechanism, can account for our observations. In any case, our data indicates that AS1 does not appear to directly modulate the molecular targets of anxiolytic drugs.

Casting a wider net via PDSP screening likewise did not reveal AS1’s site of action. Intriguingly, only two out of a panel of 45 proteins associated with neurotransmission showed even weak/moderate interactions with AS1 via radioligand binding assays. Interrogating these two receptors, the 5-HT_2B_ serotonin receptor and the sigma-1 receptor, with thermal preference behavioral assays suggested that they are similarly unlikely to mediate the effects of AS1, as neither inhibition nor activation replicated or reversed AS1’s unique behavioral effects.

That AS1 does not appear to act directly upon serotonin, dopamine, opioid, adrenergic, or acetylcholine receptors, nor some of their transporter proteins, is very intriguing and suggests that AS1 may be acting upon an unconventional molecular target. Additionally, despite its profound effect upon valence, AS1 itself is neither aversive nor attractive, only influencing behavior in the context of aversive stimuli. In this, AS1 is very unusual. To the best of our knowledge, no other known drug behaves in such a manner.

Given such unique behavior, we examined both peripheral and central neuronal activity following exposure to AS1 and noxious heat to determine in an unbiased manner which populations were most influenced by this small molecule in the hope that this would reveal some indication of its mechanism of action. While we established that AS1 had no effect upon peripheral trigeminal neuronal activity either alone or during simultaneous application of noxious heat, our pERK assay revealed a heightened neuronal activity in brain areas enriched with dopaminergic neuronal populations as well as neurons that receive dopaminergic innervation and/or are homologous to mammalian reward circuits. There are several distinct populations of dopaminergic neurons in the zebrafish forebrain, most notably in the olfactory bulb (OB), subpallium, pretectum, preoptic area, retina, and hypothalamus (which includes seven different groups, DC1-7, analogous to the diencephalic A11 dopaminergic neurons in mammals) [37, 38, 73]. Our MAP-Mapping analyses revealed that three of these (olfactory bulb, subpallial, and DC3) were among the top 25 most highly active regions that emerged when AS1-treated larval zebrafish were exposed to noxious heat (37.5 °C). While the MAP-Mapping pipeline did not definitively classify them as “dopaminergic”, it is possible that other highly active regions are also representative of dopaminergic neuron clusters based upon their geographic location and expression of certain genes. For example, the diencephalic otpb clusters 2 and 4 partially overlap with DC1, as does the diencephalic Isl1 cluster 2 [62, 74]. Of these dopaminergic clusters, the subpallial cluster was of particular interest to us, given that this portion of the telencephalon contains nuclei homologous to mammalian basal ganglia, including the nucleus accumbens, which is heavily involved in reward processing [66]. In mammals, the ventral tegmental area (VTA) in the midbrain is a major source of dopaminergic innervation to a variety of different brain regions involved in reward/valence processing, including the nucleus accumbens. While zebrafish do not possess a precise VTA homolog and do not have midbrain/mesencephalic dopaminergic neurons, the bulk of dopaminergic innervation to the subpallium is local, implying that dopaminergic neuronal clusters within this region may constitute the primary site of the teleost VTA [73]. Additionally, tracing studies have also shown that a minority of diencephalic dopamine neurons located within clusters DC2 and DC4 send projections into forebrain subpallial regions, suggesting that these neurons may also play a VTA-like role [65, 75, 76]. Interestingly, DC2 and DC4 were also moderately upregulated in zebrafish exposed to noxious heat and AS1.

Other subpallial regions also stood out in our analyses. In fact, the telencephalic Isl1 clusters 1 and 2, both located in the subpallium, exhibited the highest activity in brains of larval zebrafish concurrently treated with AS1 and noxious heat [62]. In zebrafish, telencephalic Isl1 marks striatum, pallidum, and pallidally derived septum; Isl1 + neurons of the dorsal subpallium in particular are considered part of the telencephalic basal ganglia, which in adult fish includes homologs for the caudoputamen, pallidum, and possibly nucleus accumbens [64, 65]. The next five most highly upregulated brain regions are also subpallial, and include the otpb, S1181t, and Gad1b clusters, the eminentia thalami, and the anterior commissure. While the precise identity and function of some of these regions is unknown (e.g., the otpb and S118t clusters), others are more clearly associated with zebrafish basal ganglia and amygdala equivalents. For example, the eminentia thalami, or thalamic/prethalamic eminence, gives rise to the ventral entopeduncular nucleus, a basal ganglia nucleus conserved in mammals, and a major source of afferents/projections into the habenulae, a set of bilateral nuclei that are implicated in a wide range of behaviors, including modulation of fear and aversion as well as regulation of monoaminergic activity [42]. Additionally, the anterior commissure is a forebrain white matter tract located within the telencephalon through which approximately 40–60% of subpallial dopaminergic neurons send their inputs [73]. The subpallial Gad1b cluster may be part of the zebrafish equivalent of the central amygdala, proposed to consist of subpallial Isl1- GABAergic neurons [63]. In mammals, central amygdala circuits underlie appetitive behaviors and valence assignment, and in zebrafish, amygdala-like areas have been implicated in emotional and motivated behaviors [6, 9, 44].

Taken to together with our data suggesting that AS1 activates dopamine neurons in the context of aversive heat, the behavioral experiments in which we pharmacologically activated or inhibited dopamine receptors confirmed that this circuitry, and in particular D1R circuitry, played a significant role in mediating the effects of AS1. Whereas activation or blockade of D2 receptors had little effect upon AS1-induced attraction to aversive stimuli, treatment with D1R agonists and antagonists alike was able to attenuate the effects of AS1 in all of the behavioral assays we tested. Interestingly, neither of the D1R-targeting drugs we employed was able to completely restore aversion, even at the highest concentrations tested. In all of our behavioral assays, SCH23390 and SKF82958 generally reduced or eliminated preference for the aversive stimulus (noxious heat, AITC, the dark), but did not restore preference for the control stimulus. (The only exception being that SKF82958 did modestly restore light preference at high concentrations, but never to control levels.) Additionally, while treatment with these drugs was able to reduce the heightened swimming velocity in the 28.5 °C zone in the thermal preference assays (perhaps indicating that this region was no longer “undesirable” for larval zebrafish), swimming velocity in the 37.5 °C zone was never elevated back to baseline levels, perhaps indicating that this zone was, if not attractive, then still not aversive. This was expected with SKF82958, as unilateral activation of D1 receptors should mask the effects of context-specific dopamine release following the concurrent application of AS1 and a noxious stimulus. Blockade of D1 receptors, by contrast, could feasibly completely restore aversion, and the fact that we did not observe this is quite intriguing. This suggests that the analgesic and valence-reversing properties of AS1 may act via different mechanisms and that the latter might be mediated via D1 dopamine receptors. Alternatively, we may be unable to effectively titrate the balance between AS1 and SCH23390 pharmacologically. It is possible that application of this antagonist does not completely block D1 receptors, or at least not at the levels required to offset the effects of AS1, even at very high concentrations.

In mammals, dopamine release from the mesolimbic system has long been associated with reward and providing positive valence for pleasurable stimuli, and such dopamine release is repressed in the presence of noxious stimuli, driving activation of circuits that promote aversion [7, 18–20]. That D1R circuitry was specifically implicated in motivational valence assignment has some precedence in mammalian systems—for example, activation of D1R+ neurons in both the nucleus accumbens and the olfactory bulb of mice has been able to elicit real-time place preference, while stimulating D2R+ neurons can lead to aversion [77–79]. D1R+ neurons have also been implicated in previous reports of valence reversal/valence shifts following manipulations of reward circuitry. In rodents, a population of GABAergic striatal medium spiny neurons that co-express D1R and the melanocortin 4 receptor (MC4R) has been proposed to be critical for establishing generalized aversion. This inhibitory population is innervated by proopiomelanocortin (POMC) expressing neurons in the arcuate nucleus of the hypothalamus, and projects to midbrain dopaminergic neurons [7, 56]. Under ordinary circumstances, noxious stimuli activate POMC neurons, which leads to activation of MC4R neurons and thus inhibition of dopaminergic neurons and a reduction in dopamine release [7, 56]. Interestingly, disruption of this circuit (e.g., by genetic deletion of the MC4 receptor) was reported to cause normally threatening stimuli to become attractive, likely a result of elevated dopamine levels that ensue when this inhibitory pressure is released [7, 56]. Interestingly, glutamatergic receptor blockade specifically within the nucleus accumbens shell can also elicit an appetitive to aversive hedonic shift (i.e., render appetitive stimuli aversive), further suggesting that this region is a site for valence determination [80].

Our data supports a similar model in which AS1 acts upstream of dopamine neurons to release a “brake” on dopamine release that is established by the presence of noxious stimuli. It is unlikely that AS1 acts directly upon dopamine receptors, as direct agonism/antagonism of these receptors did not replicate the effects of AS1. This was further corroborated by results from PDSP, which similarly failed to demonstrate any direct interaction between AS1 and any of five dopamine receptors (D1–D5). Additionally, we find it less likely that AS1 directly activates dopaminergic neurons, stimulating dopamine release independent of context, as AS1 itself does not appear to be inherently rewarding/attractive based upon our observations. This begs the question, however—upon which upstream neurons is AS1 acting, and what is its molecular target? At present, these are unknowns. Mammals possess a diverse array of dopaminergic neurons that are associated with different neural processes. In many cases, these populations have specific projection patterns and receive distinct inputs from other brain regions. Dopaminergic neurons within the VTA are a prime example of this functional heterogeneity within the context of encoding reward and aversion. For instance, certain dopaminergic populations project to the lateral shell of the nucleus accumbens, where dopamine release promotes reinforcement and preference, whereas other populations project to the prefrontal cortex and encode aversion [81–83]. These populations receive discrete excitatory inputs from other brain regions (e.g., laterodorsal tegmentum, lateral habenula) as well as local inhibition from GABAergic interneurons [84, 85]. If analogous circuits are present in larval zebrafish, this complex microcircuitry offers several potential targets for AS1. It is possible that AS1 acts to inhibit local GABAergic interneurons within a VTA equivalent; AS1 may also potentially act further upstream, perhaps silencing the excitatory inputs to inhibitory neurons. While our data suggest that the MC4R pathway is unlikely to be a target of AS1 in the zebrafish, it is possible that AS1 is acting via an analogous mechanism to inhibit similar neurons that would normally suppress dopamine release in the context of noxious stimuli [7, 56].

It is also intriguing that AS1 induces attraction towards noxious (and sometimes lethal) stimuli in an intensity-dependent manner. While the exact molecular/neuronal target of AS1 upstream of dopamine release is unknown, one could speculate that a few different scenarios may account for this observation. Our experiments with both thermal and light/dark preference assays could suggest a feedback loop in which the intensity of the “brake” upon dopamine release depends upon the intensity of the noxious stimulus. It is possible that AS1 interacts with whatever neuronal population is responsible for encoding the intensity in such a way that a stronger nociceptive/aversive drive will likewise lead to a stronger release of our postulated brake. If in the larval zebrafish brain there is a tonic/baseline release of dopamine that can be dialed up or down to promote attraction or aversion, then a greater release of the brake could promote greater attraction. Alternatively, one could also imagine a scenario in which noxious stimuli send a strong activation drive to dopamine neurons at the same time they establish a brake. Salient (especially novel) stimuli, regardless of motivational valence, have been shown to elicit dopamine release in mammals [86, 87]. It is plausible that similar mechanisms may be at play in larval zebrafish—if these stimuli elicit dopamine release, and this release is graded depending upon stimulus intensity, then an AS1-induced release of the brake could result in greater positive valence for (and greater behavioral attraction towards) increasingly noxious stimuli. More research is however required to determine whether either of these mechanisms, or a different one entirely, underlies this observation.

It is possible that other regions projecting onto the dopaminergic areas identified by our MAP-MAPing experiments include AS1’s initial site of action. However, while extensive work has been done characterizing the location and projection patterns of dopaminergic neurons in the zebrafish, fewer studies have examined their presynaptic partners [38, 73, 76]. Examining the brain regions upregulated in the context of AS1 alone may provide some clue as to which brain regions these might be. It is plausible that any (or all) of these regions could contribute to the valence reversal effects of AS1 and that the concurrent application of this novel analgesic/valence modifying drug and noxious stimuli may modulate their activity in such a way to promote dopamine release, and thus reward.

## Conclusions

In summary, we have identified a small molecule, AS1, that reverses aversion to noxious stimuli across multiple sensory modalities, rendering even physically harmful environments attractive. We have demonstrated that AS1 acts via a unique, as of yet undescribed mechanism in order to engage or disinhibit D1 dopaminergic circuitry in the particular context of noxious stimuli. We have identified putative brain regions, most notably dopaminergic populations and the teleost equivalent of the basal ganglia, that may underlie this behavioral response. More work is certainly necessary to precisely identify the molecular and neuronal targets of AS1, which specific individual neurons are involved in mediating its effects, and whether our observations are translatable to mouse models. We hope that the use of AS1 can provide valuable insight into understanding, as well as a means to manipulate valence circuitry, in vertebrates, and that more nuanced understanding of its effects can be applied to the development of a new generation of drugs to treat both pain and other/psychiatric conditions that involve disruption of valence circuitry.

## Methods

### Experimental design

In this study, we initially sought to identify novel analgesics by screening an extensive small molecule library using a sensitized thermal preference behavioral assay in larval zebrafish. After discovering one molecule (AS1) that not only reduced thermal hyperalgesia but also elicited preference for aversive heat, we used other behavioral choice assays (examining responses to aversive chemicals and darkness) to probe whether this molecule’s effects on valence were generalizable across sensory modalities. We also used these behavioral assays in tandem with pharmacological manipulation (e.g., activating or blocking certain neuronal receptor proteins) to investigate which neural circuits were involved in mediating the effects of AS1. Neural activity in the context of AS1 application and aversive heat was assayed both with transgenic zebrafish expressing the genetically encoded calcium indicator CaMPARI as well as via immunohistochemistry to label phosphorylated ERK, a marker of neuronal activity.

### Zebrafish husbandry

Adult Zebrafish (*Danio rerio*) were raised with constant filtration, temperature control (28.5 ± 2 °C), illumination (14 h:10 h light–dark cycle, lights on at 9:00 AM), and feeding. All animals were maintained in these standard conditions, and the Institutional Animal Care and Use Committee approved all experiments. Adult zebrafish not used in behavioral experiments were bred in spawning traps (Thoren Caging Systems, Hazelton, PA) from which embryos were collected. Larval zebrafish were raised in petri dishes (Fisher Scientific, Hampton, NH) of E2 medium with no more than 50 embryos per dish at 28.5 ± 1 °C in an incubator (Fisher Scientific). Embryos were staged essentially as described [88] and kept until 6dpf.

### Chemicals

The following chemicals were procured from Millipore-Sigma (Burlington, MA): SCH23390 hydrochloride, sulpiride, sumanirole maleate, allyl isothiocyanate, and caffeine. SKF82958 hydrobromide, buspirone hydrochloride, cutamesine (SA 4503), and DMSO were purchased from Fisher Scientific (Palatine, IL). The naloxone hydrochloride was purchased from R&D Systems Inc. (a Bio-Techne brand, Minneapolis, MN). Diazepam (Hospira, Inc., Lake Forest, IL) and buprenorphine (Par Pharmaceuticals, Woodcliff Lake, NJ) were obtained from the Drug Services office at the University of Washington. All other reagent sources are noted in their respective sections. The original screening library was obtained from Chembridge (San Diego, CA). Compounds were selected from across their CNS-Set (https://chembridge.com/targeted-and-specialty-libraries/cns/) to maximize the diversity of molecules screened. All AS1 analogs were also obtained from Chembridge.

### Behavioral assays

#### Thermal preference/temperature choice assay

Thermal preference assays were performed as previously described [30]. In brief, individual, randomly selected 5–6dpf larval zebrafish were caught in 50–100 μL E2 media using a p200 micropiette equipped with specialized large orifice 200-μL pipet tips (USA Scientific, Ocala, FL) and deposited individually into wells of custom-made choice testing plates (one larva per well). These plates were made by machining 32 oval shaped, 20 mm x 8 mm arenas out of a 5 cm × 39 cm rectangle of plastic, which was bonded to 0.002 in thick aluminum shim (ShopAid, Woburn, MA) using a waterproof adhesive (DAP, Baltimore, MD). Once an entire plate was loaded with fish, the appropriate incubation solution was added. For all incubations, choice testing plates were returned to the 28.5 °C incubator. Following incubation in all experiments, the choice testing plate was transported to a dual solid-state heat/cool plate (AHP-1200°CP; TECA, Chicago, IL) and centered such that half of each arena was positioned over each side of the heat/cool plate. One side of the heat/cool plate was always maintained at rearing temperature (28.5 °C), while the temperature of the other side was adjusted according to the experiment. Locomotor behavior was recorded using a Canon high-definition video camcorder suspended at a fixed position above the choice testing plate. Each trial was 4 min in duration.

For single-incubation assays (e.g., testing single chemicals), larval zebrafish were caught in 100 μL E2 media and 100 μL of the control (2% DMSO) or test chemical at 2X concentration was added to each well to achieve the final desired concentration. Choice testing plates were placed in the 28.5 °C incubator to incubate for 10 min before the filmed trial. For double-incubation assays (e.g., testing the impact of various chemicals on the effects of AS1), zebrafish were caught in 50 μL E2 media and 50 μL of the first incubation solution (2X control or test chemical) was added to each well, and plates were incubated at 28.5 °C for 10 minutes. One hundred microliters of the second incubation solution (1X control or test chemical +/− 2X AS1) was added to each well, and the plate was incubated at 28.5 °C for another 10 minutes before beginning the filmed trial. For the sensitized thermal aversion assay in our initial drug screen, larvae were pre-incubated in the appropriate drug solutions for 10 min, and allyl isothiocyanate (AITC; Sigma) was added to achieve a final concentration of 0.5 μM AITC immediately before filming. The final concentration of each individual drug within each pool was ~ 8 μM. Regardless of experiment, the final DMSO concentration in all solutions was 1%.

#### Chemical attraction/aversion assays

Our agarose attraction/aversion assay was adapted from previously described experiments [47]. For the AITC aversion assay, AITC and DMSO were added to molten 0.8% agarose to achieve a final concentration of 100 mM AITC and 2% DMSO. For the AS1 attraction/aversion assay, AS1 and DMSO were added to 0.8% molten agar to achieve final concentrations of 50 mM and 2%, respectively. To construct the test chambers, the lids of 10 × 10 cm square petri dishes (Genesee Scientific, San Diego, CA) were lined on four sides with either the test (chemical-containing) or plain agarose (~ 300 μL per side) and allowed to solidify. For all experiments, ~ 30–40 randomly selected 6dpf zebrafish were caught with a 10-mL pipette pump (Bel-Art Products, Wayne, New Jersey) equipped with a glass wide-bored Pasteur pipet (Fisher Scientific) and deposited into a standard 10-cm diameter petri dish (Fisher Scientific). As much E2 media as possible was carefully removed using the same pipette. For single-incubation experiments, 30 mL of 1X solutions of the control or test chemical was added to each petri dish, and fish were incubated for 10 min at 28.5 °C. For double-incubation experiments, 15 mL of 1X solutions containing control (DMSO) or test chemicals was added to the larvae-containing petri dish. Following a 10-minute incubation, 15 mL of the second incubation solution (1X control or test chemical +/− 2X AS1) was added to the petri dish, and the fish were incubated for a second 10-minute block. The final concentration of DMSO in all solutions was 1%. In all double-incubation experiments, the final concentration of AS1 was 5 μM unless otherwise noted. After completion of the last incubation period, the contents of each petri dish were poured into separate agarose-lined square dishes. Swimming behavior was immediately recorded for 20 minutes using the same high-definition camcorder in the thermal preference assays.

To further confirm that AS1 was not inherently attractive/aversive, we performed a different version of the assay in which the agarose was eliminated. In this iteration, we designed custom 10 × 10 cm plates using clear resin (Formlabs, Somerville, MA) and a Form 3+ 3D printer (Formlabs). Each plate had a 9.5 × 0.5 cm trough at two opposing ends. Larval zebrafish (*N* ~ 30–40) were carefully pipetted into the middle of the plate in as little E2 media as possible. In each trial, 2 mL of the AS1 solution at the appropriate concentration was deposited into one trough, while 1% DMSO was added to the opposite trough. Just enough E2 media was added to the plate to join the small E2 pool containing larval fish to the contents of each trough, and care was taken to ensure the solution was disturbed as little as possible. Swimming behavior was then recorded for 20 minutes. In the case of 1 mM AS1, 10% DMSO was added to the control side given that that was the concentration of DMSO vehicle in that solution.

#### Light/dark preference (phototaxis) assay

In the light/dark preference assay, randomly selected 6dpf larval zebrafish (*N* ~ 30–40) were carefully pipetted onto a 10-cm square petri dish (Genesee Scientific) using a 10-mL pipette pump equipped with a glass wide-bored Pasteur pipet. As much E2 media as possible was carefully removed using the same pipet. For single-incubation experiments, 30 mL of 1X solutions of the control or test chemical was added to each petri dish, and fish were incubated for 10 min at 28.5 °C. For double-incubation experiments, 15 mL of 1X solutions containing control (DMSO) or test chemicals was added to the larvae-containing petri dish. Following a 10-minute incubation period, 15 mL of the second incubation solution (1X control or test chemical +/− 2X AS1) was added to the petri dish, and the fish were incubated for a second 10-minute block. The final concentration of DMSO in all solutions was 1%. In all double-incubation experiments, the final concentration of AS1 was 2.5 μM. These petri dishes were then positioned over a horizontally oriented computer monitor displaying a PowerPoint presentation. For standard light/dark preference assays, a blank white slide was initially presented for 1 min, after which the presentation would automatically advance to a slide in which half of the display was black. The petri dish with larvae was positioned such that exactly half was directly over the dark side, and the other half was directly over the light side. After 4 min, the presentation automatically advanced to a slide in which the black and white halves switched places. A total of five 4-min trials, with the dark/light halves automatically switching position between trials, were recorded. For the gradient phototaxis assay, experiments were performed identically, except that the “dark” half of the PowerPoint presentation was one of six shades of gray.

In all experiments, an initial still frame of video was taken during the minute where the blank slide was presented in order to quantify the total number of fish in the experiment. Following this, still frames were taken at 30-s intervals for each trial (*T* = 0, 30, 90, 120, 150, 180, 210, and 240 s), and the number of fish present in the light half of the arena was counted. To generate the graphs that looked at swimming patterns over time in a trial (e.g., Fig. 3D), the average percentage of fish in the light at each time point (*T* = 0, *T* = 30, etc.) was calculated across all five trials. Only counts from the final five time points (*T* = 120–240 s) of the last four trials were counted when calculating the average percentage of fish in the light (e.g., Fig. 3E), to allow fish to have time to make a choice.

### CaMPARI neuronal activity assay

*elavl3:CaMPARI* zebrafish in the Casper background were simultaneously exposed to chemical stimuli and a 405-nm light in order to permanently photoconvert active neurons as previously described [31, 61]. Briefly, 6dpf larval zebrafish were anesthetized with iced E2 medium, immobilized with a harp (Harvard Apparatus, Cambridge, MA), and paralyzed by injecting α-bungarotoxin protein (Invitrogen, Waltham, MA) into the chest cavity using microinjection needles pulled on a Flaming-Brown Micropipette Puller (model P-87, Sutter Instrument Co., Novato, CA) and a Picrosprizter II microinjection apparatus (General Valve Corporation, Fairfield, NJ). Paralyzed fish were then pre-incubated in either 1% DMSO or 5 μM AS1 for 2 min and then immersed in a water bath set to either rearing temperature (28.5 °C) or noxious heat (36.5 °C). Following this incubation period, fish were immediately placed glass-bottomed dishes (Wilco Wells, Netherlands) and placed on the stage of an inverted fluorescent microscope (Olympus, Japan, model Ix81S1F-3) and the larvae were exposed to a 405-nm light for 40 s using MetaMorph software (Molecular Devices, San Jose, CA). Post-exposure fish were removed from the chemical and placed in a petri dish filled with E2 media and tricaine to prevent any future activation of sensory neurons. Immediately prior to imaging, larvae were mounted on coverslips in 1.5% agarose + tricaine in E2 media. Trigeminal (TG) and surrounding neural tissue were imaged using a 20X lens on an LSM 880 confocal microscope (Zeiss, Germany). Zen Black software was used to scan through the entire TG. Images were examined for photoconverted (red-labeled) neurons, and totals were established for each TG in each condition.

### pERK immunolabeling

6dpf larval zebrafish (*N* ~ 10–20) in the Casper background were placed into 5-mL microcentrifuge tubes (VWR, Radnor, PA) with either 1% DMSO, 2.5 μM AS1, or 5 μM AS1. Depending upon the experimental condition tested, these tubes were placed in either a 28.5 or 37.5 °C water bath for 15 min [89]. Following the 15-min exposure, fish were immediately anesthetized with tricaine and fixed in 4% paraformaldehyde/0.25% Triton-X for ~ 20–24 h at 4 °C. Following fixation, antibody labeling for both total ERK (tERK) and phosphorylated ERK (pERK) was performed as previously described [89]. In brief, larval zebrafish were washed with 0.25% PBT (1X PBS with 0.25% Triton-X) 2–3 times, incubated in 150 mM Tris–HCl (pH 9) at 70 °C for 15 min, rinsed with PBT, and incubated in 0.05% Trypsin–EDTA for 45 min on ice. Samples were then incubated in blocking buffer (1X PBS, 0.3% Triton-X, 10% goat serum) at room temperature on a rocker for at least 1 h. The larvae were then incubated in a primary antibody solution (1:500 rabbit monoclonal Phospho-p44/42 MAPK (Erk1/2) (Thr202/Tyr204) (D13.14.4E) XP and 1:500 mouse monoclonal p44/42 MAPK (Erk1/2) (L34F12), Cell Signaling Technologies, Inc., Danvers, MA) at 4 °C on a rocker for up to 3 days. After this, samples were washed three times in PBT and incubated in a secondary antibody solution (AlexaFluor goat anti-mouse 488 and AlexaFluor goat anti-rabbit 568, both 1:500, Invitrogen) at 4 °C on a rocker shielded from light for 24 h. Samples were then washed 3 times in PBT and stored in 50% glycerol/1X PBS at 4 °C until imaging.

### Confocal imaging and MAP-mapping

Fixed, pERK/tERK immunolabeled zebrafish were dorsally mounted in ~ 1.5% low-melt agarose to facilitate imaging. Entire brains were imaged using a 10X air objective on a Zeiss LSM 880 confocal microscope (5 μm step size). In order to map experimental brains onto a reference brain, these composite confocal z-stacks were first split into individual channels in ImageJ, and each of those stacks was saved as an.nrrd file. Image stacks in this file format were then registered to a reference brain using the CMTK registration tool (GUI plugin courtesy of the Jefferis lab) on ImageJ [62, 90, 91]. In the CMTK registration GUI, the registration parameters were set to “Cachero, Ostrovsky 2010”, and -awr 0102 -X 52-C 8 -G 80 -R 3 -A –accuracy 0.4 -W –accuracy 1.6 were used as further registration parameters [91]. Registered stacks were then individually visually inspected to ensure that they had registered correctly, and all error-free stacks were then downsampled (“smoothed”) using a previously developed ImageJ script (PrepareStacksForMAPMapping.ijm) [62] and sorted into individual folders based upon condition. Conditions were as follows: Control (1% DMSO at 28.5 °C), AS1 Only (2.5 or 5 μM AS1 at 28.5 °C), Heat Only (1% DMSO at 37.5 °C), and AS1 + Heat (2.5 or 5 μM AS1 at 37.5 °C). Each experimental condition (AS1 Only, Heat Only, and AS1 + Heat) was then compared to the Control Group using the MakeTheMAPMap.m Matlab script. One of the output files for this script, a SignificantDeltaMedians file, was then used as an input to run the ZBrainAnalysisOfMAPMaps.m Matlab script, which generated excel files showing which regions of interest (ROIs) were significantly up- or downregulated from each comparison. Net activation for each ROI was determined by subtracting the negative signal from the positive signal. To determine neural activity specific to AS1 treatment in the context of noxious heat, the AS1 Only and Heat Only signals were subtracted from the AS1 + Heat values for each ROI.

### Statistical analyses

All statistical analyses are detailed in their respective subsections of the “Methods” section, and statistical tests for each experiment are specified in the appropriate figure legend. Experimental data obtained from the pERK imaging assays were initially analyzed using custom Matlab scripts from the aforementioned pipeline, and then exported into Excel for further processing. All statistical tests were performed using GraphPad Prism software versions 6.04 and 9.3.1 (GraphPad Software, San Diego, CA). All data are presented as means + s.e.m. unless otherwise indicated. *^/#/+^ *p* < 0.05, **^/##/++^ *p* < 0.01, ***^/###/+++^ *p* < 0.001.

### Supplementary Information

**Additional file 1:** **Database 1.** Raw data from the drug screen. Raw data from the initial drug screen performed upon the Chembridge small molecule library. The first tab (labeled KEY) contains information about all of the individual drugs screened from this library, including data pertaining to the chemical structure (i.e., chemical formula, name, and molecular weight), location within the library (i.e., plate number and coordinates within the plate), and Chembridge ID number. All subsequent tabs are labeled with the number of the specific plate that was screened (i.e., 2 corresponds to Plate 2, 3 to Plate 3, etc.). These tabs contain the percentage of time individual fish spent in the 28.5°C temperature zone for control, AITC, and drug-treated conditions, as well as summary statistics (mean, s.e.m., and N) for all of these groups. Each pool contains the eight drugs within a single column of the plate; Pool 2 corresponds to Column 2, Pool 3 to Column 3, and so on. Though the drug library was contained in 96-well plates, only Columns 2-11 of each plate contained drugs, thus Pools 1 and 12 are absent from this screen.

**Additional file 2:** **Movie 1.** Temperature Choice Assay. A representative ~30s video clip of an acute (non-sensitized) thermal preference (temperature choice) assay. The behavior plate is positioned over the hot plate such that the top half of each arena is over the 37.5°C side, while the bottom half of each arena is over the 28.5°C side. The first (leftmost) 16 larvae have been incubated in 1% DMSO for 10 min prior to (and during) the experiment, whereas the second (rightmost) 16 larvae have been incubated in 5 µM AS1. As shown, vehicle-treated larvae tend to swim on the 28.5°C side, and AS1-treated fish prefer the 37.5°C zone.

**Additional file 3:** **Supplementary Table 1.** Structural analogs of AS1 and their effects upon temperature preference. A list of the structural analogs of AS1 tested in our thermal preference assay (see Fig. 2D), their formal chemical names and structures, and whether or not they were capable of reversing preference for rearing temperature (28.5 °C) in our temperature choice assay. All compounds were obtained from ChemBridge (San Diego, CA). Compound # refers to the ChemBridge ID #. AS1 is included in the final row for purposes of comparison.

**Additional file 4:** **Movie 2.** AITC Aversion Assay, 1% DMSO. A representative ~30s video clip of vehicle (1% DMSO) treated larvae in a typical AITC aversion assay. The top edge of the square dish (marked with a red dot) has been lined with 300 µL 100 mM AITC in agarose, whereas all other edges have been lined with 300 µL of plain agarose. These control fish strongly avoid the side of the dish lined with 100 mM AITC agarose, and can be seen rapidly swimming away from this edge if they happen to approach it.

**Additional file 5:** **Movie 3.** AITC Aversion Assay, 5µM AS1. A representative ~ 30s video clip of AS1-treated (5 µM) larvae in a typical AITC aversion assay. Like Movie 2, the top edge of the square dish (marked with a red dot) has been lined with 300 µL 100 mM AITC in agarose, whereas all other edges have been lined with 300 µL of plain agarose. As shown, many larvae swim towards the edge of the square petri dish that is lined with 100 mM AITC in agarose. Fish that reach this side of the dish tend to linger, rather than immediately departing the zone like the vehicle-treated larvae shown in Movie 2.

**Additional file 6:** **Movie 4.** Phototaxis Assay. A representative video clip from a phototaxis/light-dark preference assay, comprising 16 min (four 4-min trials) of footage, sped up to 16x normal speed. As shown, larval zebrafish treated with 1% DMSO tend to congregate in the light half of the arena, and quickly migrate to the light half when the light and dark zones are flipped. Conversely, larval zebrafish treated with 2.5 µM AS1 avoid the light—when the light and dark zones are switched between trials, the larvae that are suddenly exposed to the light will quickly swim to the other half.

**Additional file 7:** **Supplementary Table 2.** Brain regions specifically upregulated in the context of 5 µM AS1 and noxious heat (37.5 °C). A complete list of all of the regions of interest (ROIs) identified from MAP-Mapping analysis of pERK/tERK-labeled brains of larval zebrafish that were incubated in 5 µM AS1 and exposed to noxious heat. The “Signal Specific to AS1 + Heat” was obtained by subtracting the net signals of the “AS1 Only” and “Heat Only” groups from the net signal of the “AS1 + Heat” group for each ROI. ROIs are ranked in order from highest to lowest signal.

**Additional file 8:** **Supplementary Table 3.** Brain regions upregulated in the context of 5 µM AS1 alone (at 28.5 °C). A complete list of all of the regions of interest (ROIs) identified from MAP-Mapping analysis of pERK/tERK-labeled brains of larval zebrafish that were incubated in 5 µM AS1 and exposed to rearing temperature (28.5 °C). The “Net Signal” was obtained by comparing the “AS1 Only” group to the control group, and subtracting the negative signal from the positive signal. ROIs are ranked in order from highest to lowest signal.

**Additional file 9:** **Supplementary Table 4.** Brain regions upregulated in the context of heat alone (37.5 °C). A complete list of all of the regions of interest (ROIs) identified from MAP-Mapping analysis of pERK/tERK-labeled brains of larval zebrafish that were incubated in 1% DMSO and exposed to noxious heat (37.5 °C). The “Net Signal” was obtained by comparing the “Heat Only” group to the control group, and subtracting the negative signal from the positive signal. ROIs are ranked in order from highest to lowest signal.

**Additional file 10:** **Supplementary Table 5.** Brain regions specifically upregulated in the context of 2.5 µM AS1, both alone (at 28.5 °C) and under conditions of noxious heat (37.5 °C). The same information shown in Additional files 7 and 8, but for experiments utilizing 2.5 µM AS1. A complete list of all of the regions of interest (ROIs) identified from MAP-Mapping analysis of pERK/tERK-labeled brains of larval zebrafish that were incubated in 2.5 µM AS1 and exposed to noxious heat. The “Signal Specific to AS1 + Heat” was obtained by subtracting the net signal of the “AS1 Only” and “Heat Only” groups from the net signal of the “AS1 + Heat” group for each ROI. The “Net Signal in AS1 Only vs Control” was obtained by comparing the “AS1 Only” group to the control group. ROIs are ranked in order from highest to lowest signal.

## Data Availability

All processed data generated or analyzed during this study are included in this article. Raw datasets used and/or analyzed during the current study are available from the corresponding author on reasonable request.

## Abbreviations

ACC: Anterior cingulate cortex
AITC: Allyl isothiocyanate
AS1: Analgesic Screen 1
CNS: Central nervous system
Hcrt: Hypocretin
MCR4: Melanocortin 4 receptor
MOR: Mu opioid receptor
MSN: Medium spiny neuron
OB: Olfactory bulb
OXTL: Oxytocin
PDSP: Psychoactive Drug Screening Program
pERK: Phosphorylated extracellular signal-regulated kinase
POMC: Proopiomelanocortin
tERK: Total extracellular signal-regulated kinase
TRPA1: Transient receptor potential ankyrin 1
VTA: Ventral tegmental area
