# Publisher Correction: A novel small molecule, AS1, reverses the negative hedonic valence of noxious stimuli

**DOI:** 10.1186/s12915-023-01634-x

**Published:** 2023-06-06

**Authors:** Kali Esancy, Lais L. Conceicao, Andrew Curtright, Thanh Tran, Logan Condon, Bryce Lecamp, Ajay Dhaka

**Affiliations:** 1 Department of Biological Structure, University of Washington, Seattle, USA; 2 Graduate Program in Neuroscience, University of Washington, Seattle, USA

**Publisher Correction: BMC Biology 21, 69 (2023)**

**https://doi.org/10.1186/s12915-023-01573-7**

The original article [1] erroneously presented a duplicate of Additional File 2 and omitted Additional File 6 due to a technical error on the publisher’s side. The affected Additional Files have since been amended.
